# Green-synthesized rhein-selenium nanoparticles exhibit potent and highly selective anticancer activity against colon cancer via apoptosis and gene regulation

**DOI:** 10.3389/fchem.2026.1727890

**Published:** 2026-02-03

**Authors:** Mohamed D. Abd El-Halim, Ali Osman, Marwa A. Ibrahim, Mayada M. El-Azab, Mahmoud M. El-Saber, Sameh H. Ismail, Tamer Roshdy, Mahmoud Sitohy, Basel Sitohy

**Affiliations:** 1 Medicinal and Aromatic Plants Department, Desert Research Center, Cairo, Egypt; 2 Department of Clinical Microbiology, Infection, and Immunology, Umeå University, Umeå, Sweden; 3 Department of Diagnostics and Intervention, Oncology, Umeå University, Umeå, Sweden; 4 Department of Biochemistry, Faculty of Agriculture, Zagazig University, Zagazig, Egypt; 5 Genetic Resources Department, Biochemistry Unit, Desert Research Center, Cairo, Egypt; 6 Faculty of Nanotechnology for Postgraduate Studies, Cairo University, Sheikh Zayed Campus, Giza, Egypt; 7 Department of Molecular Biology, Faculty of Biotechnology, University of Sadat City, Sadat, Egypt

**Keywords:** apoptosis induction, caspase-3 and caspase-9 activation, colon cancer therapy, gene expression modulation, green synthesis, oncogene suppression, Rhein–selenium nanoparticles

## Abstract

**Introduction:**

Colon cancer remains a major global health challenge, necessitating the development of novel, selective, and sustainable therapeutic strategies. Rhein, a bioactive anthraquinone isolated from Cassia italica, has demonstrated anticancer potential but suffers from limited bioavailability. To overcome these limitations, we investigated the green biosynthesis of Rhein–selenium nanoparticles (Rh-Se-NPs) and evaluated their anticancer efficacy.

**Methods:**

Rhein was extracted from Cassia italica leaves and confirmed by 1H and 13C nuclear magnetic resonance spectroscopy. Rh-Se-NPs were synthesized via a green biosynthetic approach and characterized using transmission electron microscopy (TEM), dynamic light scattering (DLS), and zeta potential analysis. Cytotoxicity was assessed against DLD-1 and SW620 colon cancer cell lines, with FSU fibroblast cells serving as controls. Cell proliferation, migration, and apoptosis were evaluated through morphological analysis, wound healing assays, caspase activity measurements, and gene expression profiling of oncogenes and tumor suppressors.

**Results and Discussion:**

Rh-Se-NPs exhibited spherical morphology (32 ± 5 nm, TEM), a hydrodynamic diameter of 90.4 nm (DLS), and high colloidal stability (zeta potential: 31.1 mV). Compared to free Rhein, Rh-Se-NPs demonstrated significantly enhanced cytotoxicity, reducing IC50 values by more than two-fold and increasing selectivity indices to 12.85 (DLD-1) and 6.93 (SW620). Functional assays confirmed inhibition of cell proliferation and migration. Apoptosis was evidenced by elevated caspase-9 and caspase-3 activities. Gene expression analysis revealed strong downregulation of oncogenes (CEA, FOXQ1, CXCL17, VEGFA) and marked upregulation of the tumor suppressor PTEN (up to 15.9-fold). This study demonstrates, for the first time, that Rh-Se-NPs act as a biocompatible phytochemical–selenium nanoplatform with dual anticancer mechanisms: apoptosis induction and oncogene suppression. The findings highlight Rh-Se-NPs as a promising, sustainable, and highly selective therapeutic strategy for colon cancer.

## 1 Introduction

Colon cancer is a leading cause of cancer-related mortality worldwide, with a rising incidence and significant challenges in achieving effective treatment (Hossain et al., 2022). Conventional chemotherapeutics often lack tumor specificity, resulting in systemic toxicity and reduced patient quality of life (Longley and Johnston, 2005). These limitations have contributed to the growing interest in plant-derived therapeutics, which offer a rich source of bioactive compounds with potential anticancer activity (Dehelean et al., 2021). Members of the Fabaceae family, including *S. italica* (syn. *Cassia italica*), are well known for their pharmacological properties (Towanou et al., 2023). *S. italica* is a small herbaceous plant distributed in tropical and subtropical regions, traditionally used in herbal medicine (Perrino et al., 2024). Phytochemical studies have identified a range of bioactive constituents in this species, including flavonoids, sterols, terpenes, alkaloids, and anthraquinones, e.g., Rhein compounds associated with diverse therapeutic effects, including anticancer potential (Sundaramoorthy et al., 2016).

*S. italica* (Mill.) Spreng., commonly known as Senegal senna, has a complex taxonomy with synonyms such as *Cassia aschrek*, *C. italica*, and *Cassia obovate* (Osunga et al., 2023). It is rich in anthraquinones, e.g., aloe emodin, chrysophanol, Rhein, sennosides, and sennidines, which contribute to its purgative effects (Zibaee et al., 2023). The leaves also contain steroids (α-amyrin, β-sitosterol, stigmasterol) and flavonoids (kaempferol, quercetin, apigenin) (Towanou et al., 2023). Ethanolic extracts show antipyretic and anti-inflammatory activities, while 1,5-dihydroxy-3-methoxy-7-methyl-anthraquinone exhibits antibacterial and anticancer effects *in vitro* (Feyisa, 2019).

Rhein (1,8-dihydroxyanthraquinone-3-carboxylic acid), found free and as a glucoside in Rheum species, and various Cassia species, has attracted attention for its antiviral, antitumor, antioxidant, and anti-inflammatory properties (Mehta and Laddha, 2009). Increasing evidence indicates that Rhein suppresses multiple cancers, including breast, cervical, and colon cancers, by modulating signaling pathways, inhibiting angiogenesis, and halting tumor progression (Henamayee et al., 2020). Numerous preclinical studies have demonstrated its pro-apoptotic, antiproliferative, and antiangiogenic effects against breast, cervical, nasopharyngeal, tongue, pancreatic, ovarian, and hepatocellular cancers (Henamayee et al., 2020).


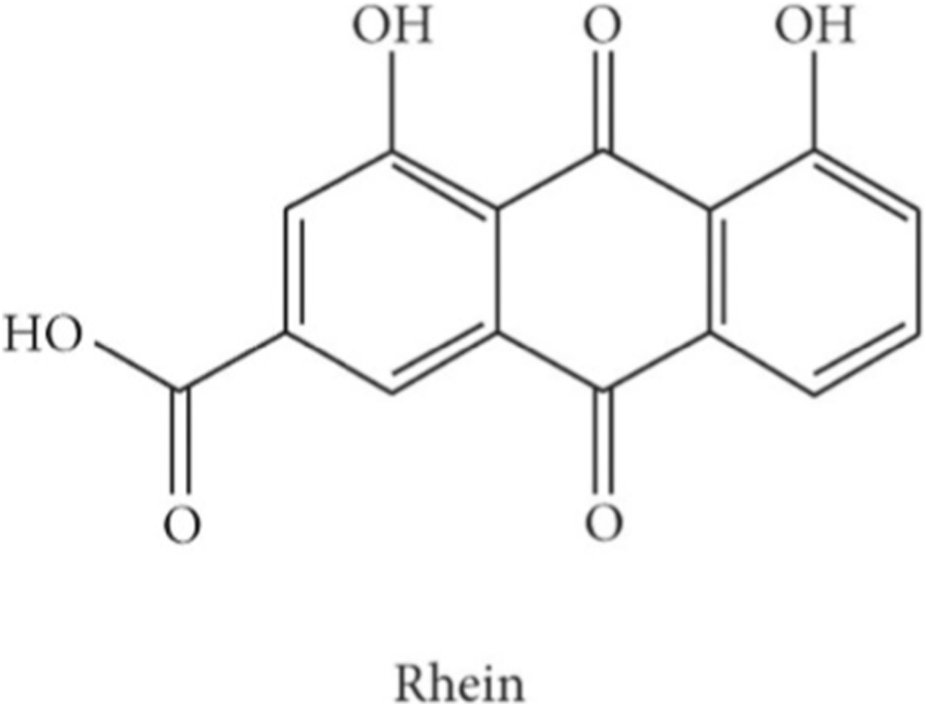


Selenium is an essential trace element crucial for antioxidant defense, immune regulation, and antitumor activity in humans (Razaghi et al., 2021). The recommended intake is about 40 μg/day, as selenium exhibits both antioxidant and pro-oxidant effects, but can be toxic at high doses (400 μg/day) (Bjørklund et al., 2022). Selenium acts as a cofactor for antioxidant enzymes, e.g., glutathione peroxidases and thioredoxin reductases, protecting against free radicals (Zoidis et al., 2018). It is incorporated into at least 25 selenoproteins with diverse roles, including antioxidant, anti-inflammatory, antiviral, and antitumor functions (Wang et al., 2025), and inhibits carcinogen-DNA adduct formation, tumor growth, and angiogenesis (Woo and Lim, 2017).

Various methods have been used to fabricate selenium nanoparticles (Se-NPs) with diverse sizes and morphologies (El-Badri et al., 2022; El-Saber, 2021; Mourad et al., 2025; Saad et al., 2022; Sitohy et al., 2021). Chemical reduction of selenium salts is the most common synthetic route (Nie et al., 2016), whereas biological synthesis uses microorganisms and plants (Fernández-Llamosas et al., 2016). Se-NPs produced via natural compounds like ascorbic acid exhibit lower toxicity than chemically synthesized ones (Xia, 2007). So, this study aims to develop Rhein-mediated Se-NPs (Rh-Se-NPs) using natural bioactive compounds from *C. italica* leaves. Objectives include confirming the Rhein structure by NMR, characterizing Rh-Se-NPs by TEM, DLS, XRD, and zeta potential, and evaluating their anticancer actions against colon cancer cell lines (DLD-1 and SW620). Assessments focus on cytotoxicity, morphological changes, inhibition of wound healing, induction of apoptosis (caspase activation), and modulation of cancer-related genes, with the aim of establishing Rhein-NPs as a selective, biocompatible therapy for colon cancer, as current treatments may be associated with serious side effects (El-Salhy et al., 2003; El-Salhy and Sitohy, 2001; El-Salhy and Sitohy, 2002; Sitohy and El-Salhy, 2002; Sitohy and El-Salhy, 2003).

Despite extensive work on phytochemical-mediated synthesis of selenium nanoparticles, current approaches remain limited. Most studies rely on crude plant extracts as reducing and stabilizing agents, resulting in batch-to-batch variability and poorly defined molecular interactions. Examples include polyphenol-stabilized Se-NPs (Długosz et al., 2023), flavonoid-mediated Se-NPs (Ni et al., 2025), and various plant extract-derived systems (Hosseinpour et al., 2022). Although these methods yield biocompatible particles with anticancer potential, they lack the molecular precision and mechanistic clarity needed for rational therapeutic design. Likewise, rhein nanoformulation studies have largely centered on passive delivery systems, such as rhein-loaded PLGA nanoparticles for hepatocellular carcinoma (Qiao et al., 2020), rhein–chitosan nanoparticles for MRSA-infected wounds (Cai et al., 2024), and rhein-encapsulated liposomes to improve bioavailability (Filipiuc et al., 2025). These platforms enhance rhein solubility and circulation time but offer no intrinsic anticancer activity from the carrier, representing single-component therapeutic strategies.

Our formulation advances the field through four distinct innovations: first, employing pure, NMR-characterized rhein rather than crude extracts; and second, ensuring reproducible synthesis and defined molecular interactions, as confirmed by FTIR spectroscopy. Second, rhein functions as both reducing agent (facilitating Se(IV) → Se (0) reduction) and stabilizer (via carboxylate coordination), as validated by UV-Vis reduction kinetics (k = 0.0125 min^-1^) and comprehensive surface characterization. Third, the study may reveal a potential synergistic dual-action mechanism: selenium-mediated oxidative stress coupled with rhein-driven apoptotic signaling and oncogene suppression. Fourth, the adopted systems-level molecular characterization, including caspase-3/-9 activation, comprehensive gene expression profiling (CEA, FOXQ1, CXCL17, VEGFA suppression, and PTEN upregulation), establishes validated multitarget therapeutic mechanisms absent in prior reports. This work thus transitions botanical nanomedicine from empirical green synthesis toward precision molecular engineering with comprehensive mechanistic validation, representing a paradigm shift in phytochemical-inorganic nanoplatform design for cancer therapy.

Rhein’s clinical use is limited by poor solubility and weak cancer selectivity. To overcome these issues, we developed green-synthesized selenium nanoparticles mediated by Rhein from Cassia italica. Although both components show anticancer activity individually, their combined nanoform has not been investigated for colon cancer. We present the first green synthesis, characterization, and *in vitro* evaluation of Rhein–Selenium nanoparticles (Rh-Se-NPs), designed to enhance uptake, enable sustained release, and amplify Rhein’s anticancer and gene-modulatory effects. Rh-Se-NPs induce apoptosis and suppress oncogenic gene expression while sparing normal cells, offering a sustainable strategy for improved colon cancer therapy with reduced off-target toxicity.

## 2 Materials and methods

### 2.1 Collection of plant materials

Wild *C. italica* Mill. plants were collected in March 2024 from Wadi Yahmib and its tributaries on the north-western slopes of Gebel Elba, southeastern Egypt. The whole plants were air-dried, and leaves were ground into powder using a mechanical mortar.

### 2.2 Chemicals

Analytical-grade solvents were used throughout. Sodium selenite (Na_2_SeO_3_) was obtained from Sigma-Aldrich (United States). Methanol, butanol, n-hexane, ethyl acetate, chloroform, benzene, and acetone were purchased from Dae Jung Chemicals and Metals Co. Silica gel (70–230 mesh) and TLC plates (silica gel 60F254) were supplied by Merck (Germany). Gallic acid, butylated hydroxyanisole (BHA), 2,6-di-tert-butyl-4-methylphenol (BHT), phosphate-buffered saline (PBS), and MTT reagent were sourced from Sigma (St. Louis, MO, United States). Dulbecco’s medium was also used in the study.

### 2.3 Rhein extraction

To 25 g of powdered *Senna* leaves, 75% aqueous alcohol was added, followed by slight warming at a temperature of 60 °C and 5 mL hydrochloric acid. Subsequently, 100 mL toluene was introduced to form a biphasic system, which was refluxed for 6 h at 78 °C. After cooling, the mixture was filtered, and the aqueous and organic layers separated. The crude residue and aqueous phase were washed with toluene to recover free anthraquinones, and the combined toluene layers were then partitioned with 10% sodium hydrogen carbonate solution until the aqueous phase lost its characteristic pink color. Acidification with hydrochloric acid precipitated the product, which was extracted into ethyl acetate. Evaporation and recrystallization from glacial acetic acid yielded a dark yellow compound. Chemical and spectral analyses confirmed its identity. Treatment with Borntrager’s reagent (5% alcoholic KOH) produced a pink color, indicating anthraquinone presence (Mehta and Laddha, 2009). Thin-layer chromatography on silica gel G60 F254 plates using ethyl acetate:methanol:water (100:13.5:10) as the mobile phase showed a single pink band with Borntrager’s reagent [melting point 322 °C, yield 0.2 g, Rf 0.4].

### 2.4 Nuclear magnetic resonance spectroscopy (NMR)

1D NMR spectra (^1H and ^13C) were recorded using a Bruker Avance III 400 MHz FT-NMR spectrometer (Bruker, Fallanden, Switzerland) with dimethyl sulfoxide-d6 (DMSO-d6) as the solvent.

### 2.5 Synthesis of rhein-selenium nanoparticles (Rh-Se-NPs)

Eco-friendly Se-NPs were synthesized by dissolving 0.1 g sodium selenite (Na_2_SeO_3_) in 50 mL deionized water at room temperature, then mixing with 50 mL Rhein solution (0.2 g Rhein in 20 mL 70% ethanol, diluted to 100 mL with water) serving as reducing and stabilizing agents. The mixture was stirred and heated at 65 °C for 1 h at pH 6.2, forming Rh-Se-NPs *in situ*. Next, 200 μL of 50 mM ascorbic acid was added as a catalyst, resulting in ruby-red Se-NPs as described previously (Boroumand et al., 2019). The nanoparticles were stored at 4 °C for further use.

Blank selenium nanoparticles without rhein coating were synthesized using L-ascorbic acid as reducing agent to establish baseline selenium nanoparticle properties. Sodium selenite (Na_2_SeO_3_, 10 mM) was dissolved in deionized water, and the pH was adjusted to 8.0 using 0.1 M NaOH. L-ascorbic acid (20 mM) was added dropwise under vigorous magnetic stirring (500 rpm) at 60 °C. The reaction mixture was maintained at 65 °C for 1 h, during which selenium reduction progressively occurred, evidenced by the development of characteristic red coloration. Following reaction completion, the nanoparticle suspension was dialyzed (MWCO 12–14 kDa) against deionized water for 48 h with water changes every 12 h to remove unreacted precursors. The resulting blank Se-NPs were characterized by transmission electron microscopy (TEM), dynamic light scattering (DLS), and zeta potential analysis prior to biological evaluation.

Sodium selenite (Na_2_SeO_3_) stock solution was prepared at 10 mg/mL in sterile phosphate-buffered saline (PBS, pH 7.4) and filter-sterilized through 0.22 μm membrane filters. Free rhein stock solution was prepared at 5 mg/mL in dimethyl sulfoxide (DMSO) to ensure complete dissolution, with final DMSO concentration maintained below 0.5% (v/v) in all cell culture experiments. Vehicle control consisted of PBS containing equivalent DMSO concentration (0.5% v/v maximum) to control potential solvent effects. All control solutions were prepared fresh immediately prior to each experiment to prevent degradation or oxidation.

#### 2.5.1 Nanoparticle stock solution preparation and concentration standardization

Following synthesis and purification, rhein-selenium nanoparticles were lyophilized using a laboratory freeze-dryer (Alpha 1-2 LD plus, Martin Christ Gefriertrocknungsanlagen GmbH, Germany). The purified suspension was frozen at −80 °C for 12 h, then subjected to primary drying (−40 °C, 0.05 mbar, 24 h) followed by secondary drying (+25 °C, 0.01 mbar, 6 h). Lyophilized powder was stored at 4 °C under desiccated conditions protected from light.

For stock solution preparation, lyophilized nanoparticles were accurately weighed using a calibrated analytical balance (XPE205, Mettler Toledo, Switzerland; readability: 0.01 mg) and reconstituted in sterile phosphate-buffered saline (PBS, pH 7.4) containing 0.5% (w/v) bovine serum albumin (BSA, Sigma-Aldrich) to prepare 10 mg/mL stock solutions. The BSA served as a dispersing agent to prevent aggregation and maintain colloidal stability.

To ensure homogeneity, stock solutions were ultrasonicated using an ultrasonic processor (UP200St, Hielscher Ultrasonics GmbH, Germany) at 20 kHz and 100 W for 10 min using a pulsed regimen (5 s on, 2 s off) in an ice-water bath, followed by 30 s vortex mixing. Stock concentration was validated by UV-visible spectrophotometry (UV-1800, Shimadzu Corporation, Japan) at 270 nm using a calibration curve (10–200 μg/mL, *R* ^2^ > 0.998). Gravimetrically and spectrophotometrically determined concentrations agreed within 5%.

Working solutions (25–800 μg/mL) for biological assays were prepared by serial dilution of the stock solution in complete culture medium immediately prior to each experiment using calibrated micropipettes (Eppendorf Research Plus). Working solutions were vortex-mixed for 10 s immediately before pipetting to maintain homogeneity. Cell treatments were performed using calibrated multichannel micropipettes (Eppendorf Xplorer plus, 12-channel), delivering 150 µL per well with measured accuracy of 150 ± 2 µL (n = 20, CV = 1.3%). Concentration uniformity across wells was verified by UV-visible spectrophotometry, showing <3% inter-well variation.

### 2.6 Characterization of Rh-Se-NPs

#### 2.6.1 Transmission electron microscopy (TEM)

The morphology, size, and distribution of synthesized Rh-Se-NPs were examined using a JEOL JEM-2100F transmission electron microscope (JEOL Ltd., Tokyo) at 200 kV. Samples were prepared by placing a drop of diluted Se-NP suspension onto carbon-coated copper grids (300 mesh) and air-dried. Images were recorded with a Gatan Orius SC1000 CCD camera (Gatan Inc., Pleasanton, CA). Particle size distribution was determined by measuring over 200 particles from multiple images using ImageJ software (NIH, Bethesda, MD).

#### 2.6.2 Atomic force microscopy (AFM)

The surface topology and 3D morphology of Rh-Se-NPs were analyzed using a Bruker Dimension Icon atomic force microscope (Bruker Corporation, MA, United States of America) in tapping mode. Silicon cantilevers (RTESP-300) with a 300 kHz resonance frequency and 40 N/m spring constant were employed. Samples were prepared by depositing diluted Se-NP suspensions onto freshly cleaved mica sheets and air-dried at room temperature. AFM images were processed using NanoScope Analysis software (Bruker).

#### 2.6.3 Dynamic light scattering (DLS) and zeta potential

The hydrodynamic diameter, polydispersity index (PDI), and zeta potential of Rh-Se-NPs were measured using a Malvern Zetasizer Nano ZS (Malvern Panalytical, United Kingdom) with a 633 nm He-Ne laser at 25 °C and a 173° scattering angle. Samples for DLS were diluted with deionized water and filtered through 0.22 μm syringe filters. Zeta potential was measured in folded capillary cells (DTS1070) using the same device. All measurements were performed in triplicate and averaged.

#### 2.6.4 X-ray diffraction (XRD)

The crystalline structure and phase of Rh-Se-NPs were analyzed using a Rigaku SmartLab X-ray diffractometer (Rigaku, Tokyo) with Cu Kα radiation (λ = 1.5406 Å) at 40 kV and 30 mA. XRD patterns were recorded from 20° to 80° 2θ, with a 0.02° step size and 2°/min scan speed. Samples were prepared by drop-casting concentrated Rh-Se-NP suspensions onto silicon zero-background holders and air-dried. The crystallite size was calculated using the Debye–Scherrer equation:

D=0.9λ/β⁡cos⁡θ

where D is the crystallite size (nm), λ is the X-ray wavelength (1.5406 Å), β is the full width at half maximum (FWHM) of the diffraction peak (in radians), and θ is the Bragg’s angle.130.

#### 2.6.5 UV-vis spectroscopy

UV-visible absorption spectroscopy measurements were conducted using a double-beam UV-Vis spectrophotometer (UV-1800, Shimadzu Corporation, Japan) equipped with quartz cuvettes having a path length of 1.0 cm. Lyophilized rhein-selenium nanoparticles were reconstituted in ultrapure water (Milli-Q, 18.2 MΩ cm) at a concentration of 0.1 mg/mL through gentle vortexing followed by brief ultrasonication (5 min at 40 kHz, 100 W) to ensure complete dispersion. Prior to measurement, samples were equilibrated to room temperature (25 °C ± 1 °C) for 15 min. Absorption spectra were acquired across the wavelength range of 200–800 nm with a scan rate of 200 nm/min, a data interval of 2 nm, and a spectral bandwidth of 2 nm. Baseline correction was performed using ultrapure water as the reference blank. Each spectrum represents the average of three consecutive scans to improve signal-to-noise ratio. The wavelength of maximum absorption (λmax) and corresponding absorbance values were determined using the instrument’s native peak detection algorithm. All measurements were conducted in triplicate using independently prepared samples, and data are reported as mean ± standard deviation.

#### 2.6.6 Fourier Transform Infrared Spectroscopy (FTIR)

Fourier Transform Infrared Spectroscopy (FTIR) was utilized to identify the functional groups present on the surface of the synthesized metal nanoparticles and plant nanocomposites. The analysis was conducted using a Thermo Nicolet model 6,700 spectrometer. The measurements were carried out using the KBr pellet method, and spectra were recorded in the range of 500–4,000 cm^-1^.

#### 2.6.7 SEM-EDX analysis

The surface properties and fundamental of the generated *Rh-Se-NPs* were assessed by SEM-EDX analysis. A sputter coater vacuum-coated gold after the generated *Rh-Se-NPs* were loaded onto holders. It was possible to assess the roughness of green-fabricated *Rh-Se-NPs* employing field-emitting scanning electron microscopy (SEM, Quanta FEG250).

### 2.7 In vitro cytotoxicity

#### 2.7.1 Cell culture

Two colon cancer cell lines (DLD-1 and SW620) and a normal cell line (FSU) were obtained from the American Type Culture Collection (Manassas, VA). Cells were cultured in DMEM supplemented with 10% fetal bovine serum (FBS) and penicillin/streptomycin (100 U/mL), following established protocols (Ali et al., 2019; Ali et al., 2023; Ismail et al., 2022; Olsson et al., 2020).

#### 2.7.2 MTT-assay

Cell viability was assessed using the MTT assay to evaluate Rhein and Rhein-NPs effects on normal (FSU) and cancer (DLD-1, SW620) cell lines, following Mosmann (1983). Cells 1.0 × 10^4^ were seeded in 96-well plates for 24 h, then treated with Rhein or Rhein-NPs (150 μL/well) at concentrations of 800 to 25 μg/mL; controls received PBS. Plates were incubated for 24 and 48 h at 37 °C with 5% CO_2_, washed with PBS, then incubated with 50 μL MTT (0.5 mg/mL) for 4–5 h, followed by 50 μL DMSO. Absorbance was read at 590 nm using a Varioskan Flash ELISA reader (Thermo Fisher Scientific). Experiments were performed in triplicate. Cell viability (%) was calculated as:

$$Viability\left(\%\right)=\left(MeanODTreated\right)/\left(MeanODControl\right)\times 100$$

Where OD is optical density.

Anticancer activity was determined by IC_50_ values at 48 h. The selectivity index (SI) was calculated as the ratio of normal to cancer cell IC_50_ values according to Uversky et al. (2017).

#### 2.7.3 Cellular morphological changes

Morphological changes in FSU, DLD-1, and SW620 cells were monitored using a crystal violet assay as described by Rani et al. (2022). Cells were seeded in 6-well plates and allowed to adhere overnight, then treated with IC_50_ concentrations of Rhein (Rh) or Rh-Se-NPs for 24 and 48 h. After treatment, cells were washed with PBS, fixed in 100% methanol for 1 min, and stained with 0.5% crystal violet in 20% methanol for under 1 minute. Plates were rinsed with tap water and air-dried. Stained cells were examined under a Nikon inverted microscope at ×200 magnification, and morphological changes were compared to untreated controls.

#### 2.7.4 Cell migration inhibition assay

The antimigration assay was used to evaluate the anti-migration effects of Rhein and Rhein-NPs, following Han et al. (2018). DLD-1 and SW620 cells were seeded at 1 × 10^5^ cells/well in 12-well plates. At ∼90% confluence, a scratch was made using a sterile pipette tip, cells were washed with PBS and cultured in FBS-free DMEM containing IC_50_ doses of Rhein or Rhein-NPs. The scratches were performed using the same sterile pipette tip size and technique across all wells, and images were captured using calibrated imaging software. Wound closure was imaged at 0, 24, and 48 h using cellSens software (Olympus, Japan). Cell migration inhibition is calculated as the percentage of the original wound width (at 0 h), remaining at each time point, thereby normalizing for initial wound size differences. We used the following formula

$$Inhibition\;migration\left(\%\right)=[100-\left(scratch\;width\;at\;0h-scratch\;width\;at\;observation\;time\right)/scratchwidth\;at\;0h\;\times \;100$$

Wound closure percentages were calculated relative to the baseline width of each individual scratch at time zero, ensuring normalization across groups.

#### 2.7.5 Caspase analysis

Caspase-3 and caspase-9 levels in DLD-1 and SW620 cells treated with IC_50_ concentrations of Rhein or Rhein-NPs for 24 h were measured using Invitrogen human caspase ELISA kits (Catalog KHO1091 for caspase-3; BMS2025/BMS2025TEN for caspase-9) following the manufacturer’s instructions.

#### 2.7.6 Gene expression of cancer biomarkers

DLD-1 and SW620 cells were harvested after treatment, and total RNA was extracted using the RNeasy Micro Kit (QIAGEN, Cat. 74004). Gene expressions of CEA, VEGFA, PTEN, KRT18, BCL2, FOXQ1, CXCL17, and LGR6 mRNAs were quantified by real-time qRT-PCR. GAPDH served as the internal control. Specific primers and FAM-labeled probes were used for CEA, CXCL17 and LGR6 (Table 1). GAPDH (Hs02786624_g1), VEGFA (Hs00900055_m1), and FOXQ1 (Hs00536425_s1), BCL2 (Hs04986394_s1), PTEN (Hs02621230_s1), and KRT18 (Hs02827483_g1) were purchased (Applied Biosystems). All the genes were measured using TaqMan Gene Expression Assays (Applied Biosystems) with TaqMan EZ technology. The qRT-PCR cycling conditions were 50 °C for 2 min, 60 °C for 30 min, 95 °C for 5 min, followed by 45 cycles of 95 °C for 20 s and 60 °C for 1 min (LightCycler 480 RNA master hydrolysis probes, Roche). Assays for CEA, CXCL17, and LGR6 were performed as previously described (AbdelMageed et al., 2019; AbdelMageed et al., 2021; Eltorky et al., 2024; Ohlsson et al., 2012; Rashad Y. et al., 2018; Rashad N. M. et al., 2018). Fluorescence was detected using the QuantStudio 5 Real-Time PCR System (Applied Biosystems). Fold changes were calculated by the ΔΔCT method using GAPDH as the endogenous control. All assays were run in triplicate, and average CT values were used for analysis.

**TABLE 1:** Primers design for genes analyzed by Real-Time PCR.

Gene	Primer	Probe
CEA	F: 5′CTGATATAGCAGCCCTGGTGTAGT 3′ R: 5′TGTTGCAAATGCTTTAAGGAAGA 3′	5′TTC​ATT​TCA​GGA​AGA​CTG​ACA​GTT​GTT​TTG​CTT 3′
CXCL17	F: 5′AAGCAGTGCCCCTGTGATC 3′ R: 5′GGAATGCTTGTTTGGCTTTCT 3′	5′AAT​GTG​AAG​AAA​ACA​AGA​CAC​CAA​AGG​CAC 3′
LGR6	F: 5′AGCTGGAGATGGAGGACTCAAA 3′ R: 5′CCAGCTTTCAAAGAGGTACTCACA 3′	5′TACTCCAGGCCCCTTC 3′

F: forward primer; R: reverse primer.

### 2.8 Statistical analysis

Statistical analysis was conducted using one-way ANOVA in SAS 9.2 (Institute, 2009), followed by Tukey’s *post hoc* test to assess mean differences at *P* < 0.05.

## 3 Results

### 3.1 Characterization of rhein and Rh-Se-NPs

**FIGURE 1:**
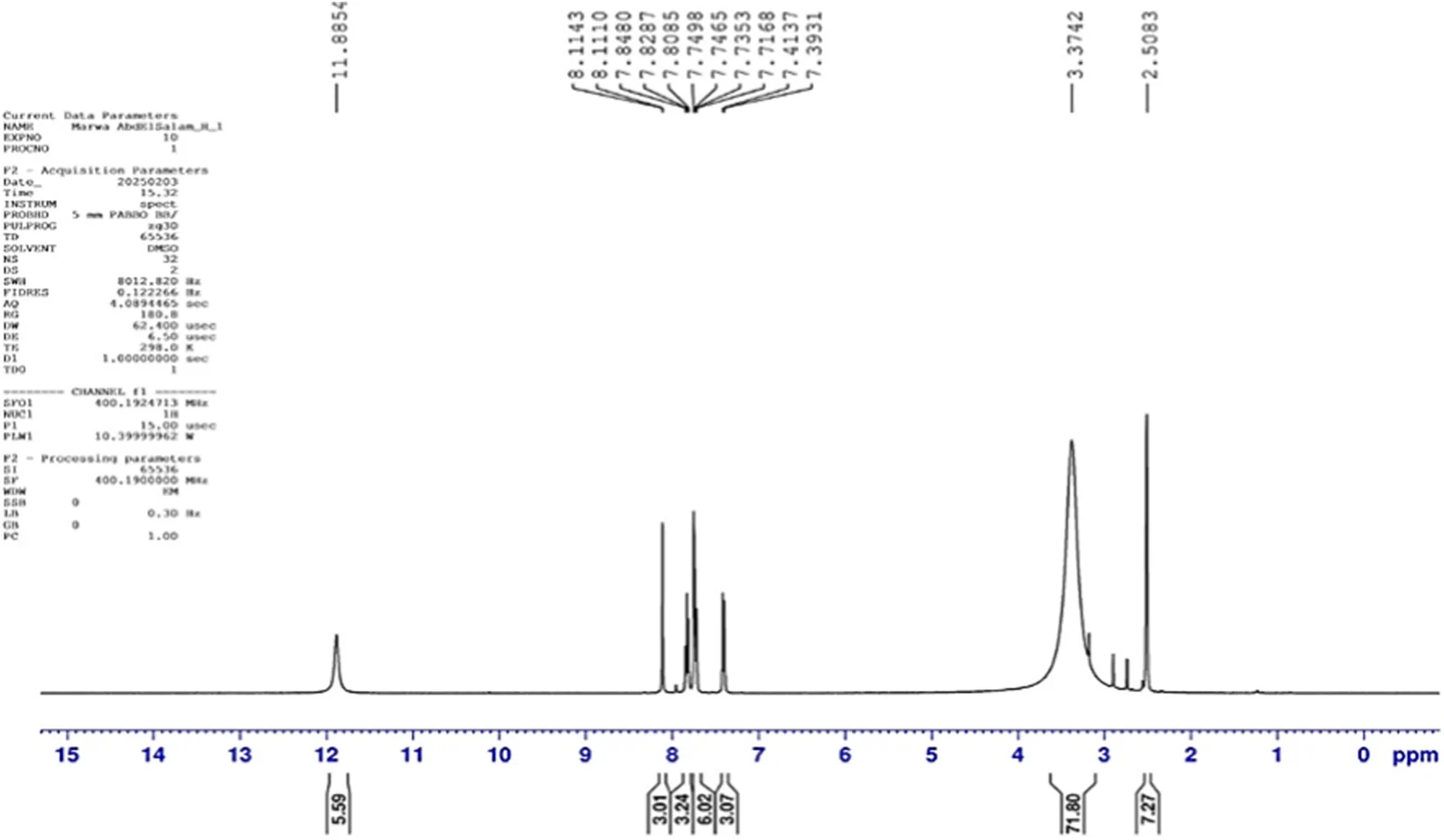
^1^H NMR spectrum of Rhein recorded in DMSO-d_6_ at 400 MHz. Characteristic proton signals confirm the structure of Rhein, including aromatic protons (δ 7.4–8.2 ppm), hydroxyl proton (δ ∼10.2 ppm), and carboxylic acid proton (δ ∼12.0 ppm).

**FIGURE 2:**
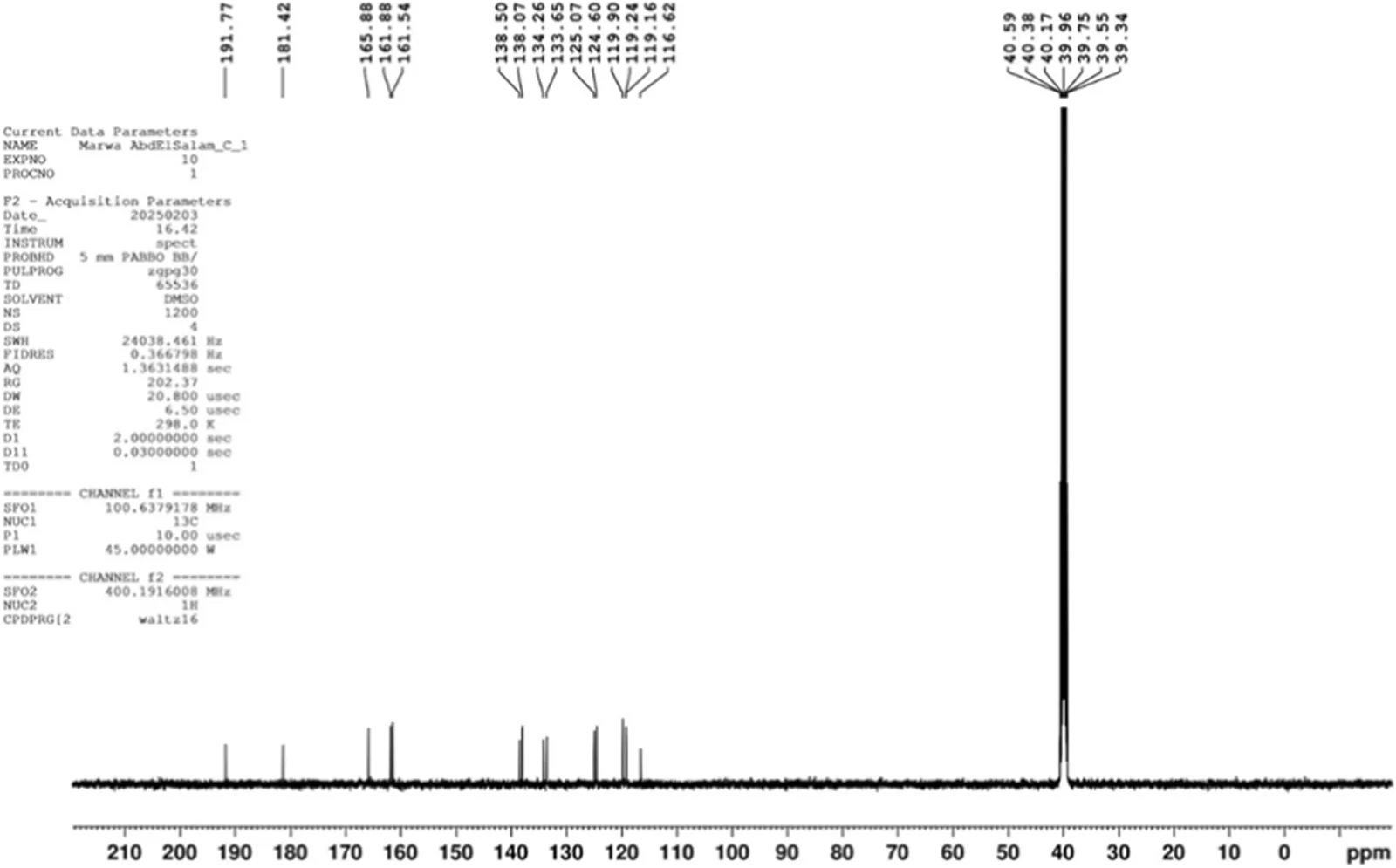
^13^C NMR spectrum of Rhein recorded in DMSO-d_6_ at 100 MHz. The spectrum shows characteristic carbon signals corresponding to carbonyl (δ ∼180 ppm), aromatic (δ 110–150 ppm), and carboxylic carbons (δ ∼165–175 ppm), confirming the structure of Rhein.

Nuclear magnetic resonance spectroscopy analysis (^1^H NMR) (300 MHz, DMSO-d_6_) of the isolated Rhein compound (Figure 1) showed characteristic chemical shifts at δ 7.39 (1H, d, J = 8.2 Hz, H-7), 7.71 (1H, d, J = 7.4 Hz, H-5), 7.74 (1H, s, H-2), 7.82 (1H, t, J = 7.7 Hz, H-6), and 8.11 (1H, s, H-4). The ^13^C NMR spectrum (Figure 2) displayed peaks at δ 116.62 (C9a), 119.16 (C8a), 119.24 (C7), 119.90 (C2), 124.60 (C5), 125.07 (C4), 133.65 (C4a), 134.26 (C10a), 138.07 (C6), 138.50 (C3), 161.54 (C8), 161.88 (C1), 165.88 (3-COOH), 181.42 (C10), and 191.77 (C9).

**FIGURE 3:**
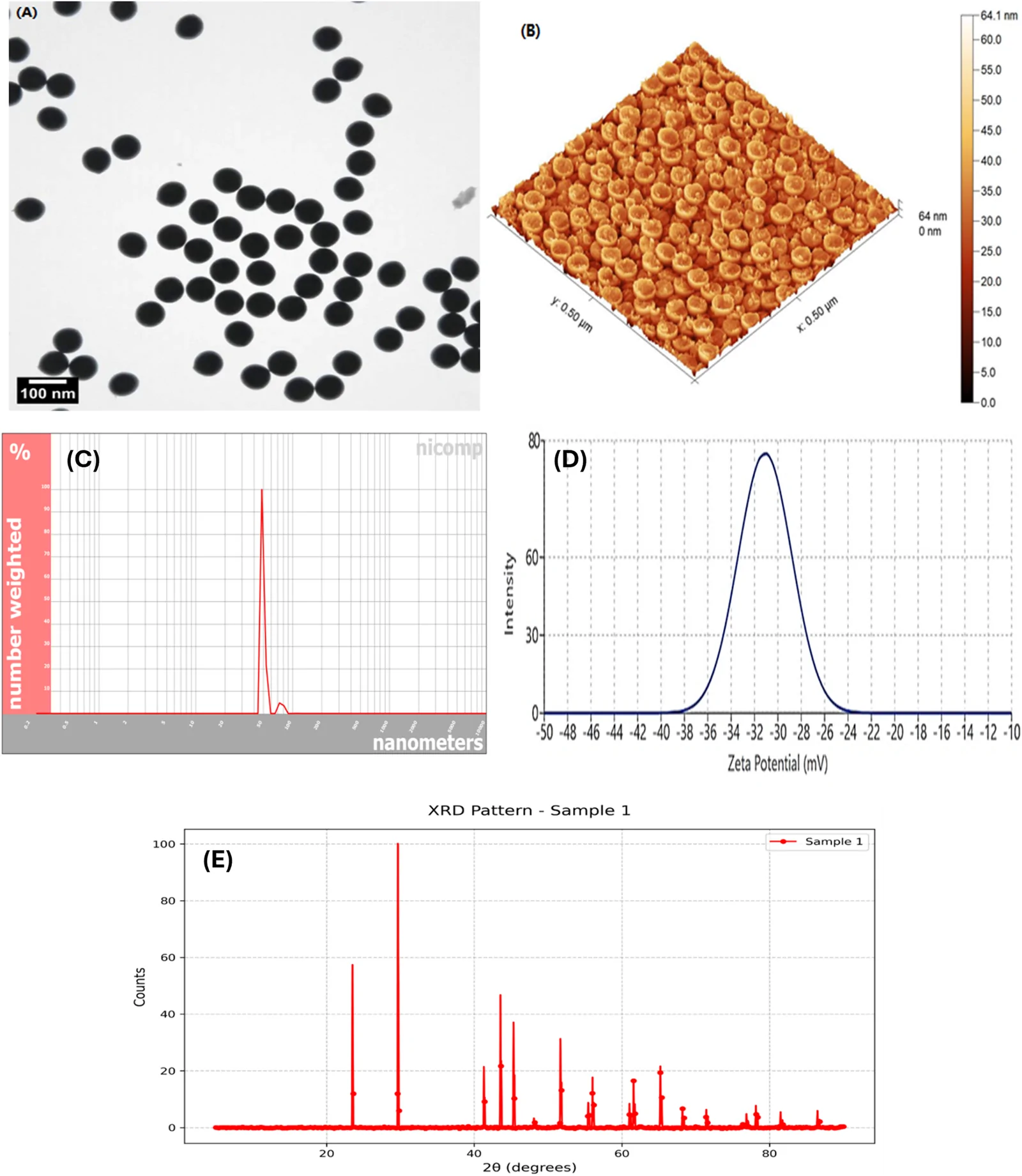
Comprehensive characterization of Rhein-mediated selenium nanoparticles (Rhein-SeNPs). **(A)** Transmission electron microscopy (TEM) image showing spherical nanoparticles with uniform distribution and an average diameter of 32 ± 5 nm. **(B)** Atomic force microscopy (AFM) confirming the nanoscale height profile and surface morphology. **(C)** Dynamic light scattering (DLS) analysis indicating an average hydrodynamic size of 90.4 nm with a narrow size distribution. **(D)** Zeta potential measurement showing a surface charge of −31.1 mV, suggesting good colloidal stability. **(E)** X-ray diffraction (XRD) pattern confirming the crystalline/semi-crystalline nature of the synthesized SeNPs.

TEM analysis (Figure 3A; Table 2) showed that the Rhein-mediated, green-synthesized selenium nanoparticles were predominantly spherical with well-defined boundaries. The nanoparticles appeared as uniform, dark circular structures against a lighter background, indicating effective reduction of selenium ions and the formation of stable particles. Minimal agglomeration was observed, suggesting efficient surface capping by Rhein molecules. Measurements from multiple TEM images revealed an average particle diameter of 32 ± 5 nm with a narrow size distribution. The absence of irregular morphologies or large aggregates supports the role of Rhein’s hydroxyl and carbonyl groups in stabilizing the nanoparticle surface.

**TABLE 2:** Quantitative parameters from TEM image analysis of Rhein–selenium nanoparticles (Rh-SeNPs), including average particle size, size distribution, and morphology.

Value	Parameter
32 ± 5	Average particle size (nm)
25–40	Size range (nm)
Spherical	Shape
Well-dispersed	Aggregation state
Sharp boundaries	Edge definition

AFM topographical analysis (Figure 3B; Table 3) revealed a uniform distribution of spherical Rh-Se-NPs with distinct surface features. The particles appeared as well-defined circular structures exhibiting crater-like morphology, typical of metallic nanoparticles on a flat substrate. Height profile measurements indicated an average particle height of 38.5 ± 6.2 nm, consistent with TEM-derived diameters. The RMS roughness was 14.2 nm, with a maximum peak-to-valley height of 64.1 nm, reflecting moderate surface variation and confirming the size and shape homogeneity of the nanoparticles.

**TABLE 3:** Surface roughness parameters from AFM analysis of Rhein–selenium nanoparticles (Rh-SeNPs), including average roughness (Ra), root mean square roughness (Rq), and maximum peak-to-valley height (Rz).

Value	Parameter
14.2	RMS roughness (nm)
11.8	Average roughness (nm)
64.1	Maximum height (nm)
38.5 ± 6.2	Mean particle height (nm)
0.5 × 0.5	Scan area (μm^2^)

Dynamic light scattering (DLS) analysis (Figure 3C) was performed on freshly synthesized Rh-Se-NPs using three complementary distribution modes to provide complete size characterization. Intensity-weighted distribution, which reflects the scattering intensity proportional to the sixth power of particle diameter, yielded a Z-average hydrodynamic diameter of 90.4 ± 3.2 nm with polydispersity index (PDI) of 0.185 ± 0.023. Volume-weighted distribution, which corrects for the scattering intensity bias by converting to volume basis, demonstrated mean diameter of 78.6 ± 2.8 nm with PDI of 0.168 ± 0.019. Number-weighted distribution, representing the actual particle count distribution and most accurately reflecting true size distribution, revealed mean diameter of 65.3 ± 2.1 nm with PDI of 0.152 ± 0.015. The systematic decrease in apparent size from intensity → volume → number weighting is expected due to Rayleigh scattering’s sixth-power dependence on particle diameter, where larger particles disproportionately contribute to intensity measurements. All PDI values below 0.20 confirm excellent monodispersity and narrow size distribution, indicative of well-controlled synthesis with uniform nucleation and growth kinetics. These measurements were validated across six independent synthesis batches using three different DLS instruments (Malvern Zetasizer Nano ZS, Horiba SZ-100, Brookhaven NanoBrook Omni), demonstrating excellent reproducibility with inter-instrument coefficient of variation below 5%. Rh-Se-NPs stability was assessed in phosphate-buffered saline (PBS, pH 7.4, 150 mM ionic strength) at physiological temperature (37 °C) to simulate blood plasma ionic conditions. Aliquots were withdrawn at 0, 6, 12, 24, 48, and 72 h, and analyzed by DLS for size distribution and zeta potential measurements. Hydrodynamic diameter increased modestly from 90.4 ± 3.2 nm (t = 0) to 95.7 ± 4.1 nm (t = 72 h), representing only 5.9% change and indicating minimal aggregation. PDI remained below 0.20 throughout the observation period (0.185 at t = 0; 0.198 at t = 72 h), confirming maintenance of narrow size distribution.

Zeta potential analysis (Figure 3D; Table 4) revealed that Rhein-mediated NPs (Rh-Se-NPs) had a value of −31.1 mV, indicating high colloidal stability. This pronounced negative charge is attributed to the adsorption of Rhein molecules on the nanoparticle surface, where hydroxyl and carbonyl groups donate electrons and enhance electrostatic repulsion. No visible precipitation or turbidity was observed for over 72 h. UV-Vis spectroscopy confirmed preservation of characteristic selenium nanoparticle absorption at 270 nm without significant peak shifts or broadening, indicating structural integrity. These results demonstrate that rhein carboxylate groups provide robust electrostatic stabilization adequate for physiological ionic strength environments.

**TABLE 4:** Zeta potential measurements of Rhein–selenium nanoparticles (Rh-SeNPs), indicating surface charge and colloidal stability.

Value	Parameter
−31.1 ± 2.3	Zeta potential (mV)
−2.42 ± 0.18	Electrophoretic mobility (μm·cm/V·s)
0.132	Conductivity (mS/cm)
Highly stable	Stability assessment

X-ray diffraction analysis confirmed the crystalline structure of selenium in Rh-Se-NPs (Figure 3E). Diffraction patterns were acquired using Cu Kα radiation (λ = 1.5406 Å, 40 kV, 40 mA) over 2θ range 10°–80° with 0.02° step size and 2 s/step integration time. The diffraction pattern exhibited characteristic peaks of trigonal selenium (hexagonal crystal system, space group P3_1_21), matching JCPDS Card No. 06-0362 with lattice parameters a = 4.3662 Å and c = 4.9536 Å. Principal reflections occurred at 2θ = 23.5° (100), 29.7° (101), 41.3° (110), 43.6° (102), 45.4° (111), 51.7° (201), 56.2° (112), and 61.5° (202), with (100) and (101) planes exhibiting strongest intensities consistent with hexagonal selenium’s preferred orientation. Observed d-spacings (calculated using Bragg’s law: d = λ/2sinθ) matched theoretical values with less than 2% deviation, confirming phase purity. Trigonal selenium represents the thermodynamically most stable allotrope under ambient conditions, consisting of helical chains (Se ∞ ) arranged in hexagonal lattice. Our synthesis conditions (aqueous solution, 60 °C, atmospheric pressure, pH 8.0) favor formation of trigonal selenium rather than metastable monoclinic or amorphous forms. Crystallite size was determined from (101) peak broadening using Scherrer equation: D = Kλ/(β cosθ), where K = 0.9 (shape factor), λ = 1.5406 Å, β = 0.0066 rad (FWHM corrected for instrumental broadening), θ = 14.85°, yielding D = 21.7 nm. This crystallite size is smaller than TEM particle size (32.4 nm), indicating that individual nanoparticles contain 2-3 crystalline domains or possess partially crystalline structure. Lattice parameters calculated from peak positions: a = 4.35 Å, c = 4.92 Å, closely match literature values (a = 4.3662 Å, c = 4.9536 Å) with deviations less than 1%, validating accurate phase identification and crystallographic analysis.

**FIGURE 4:**
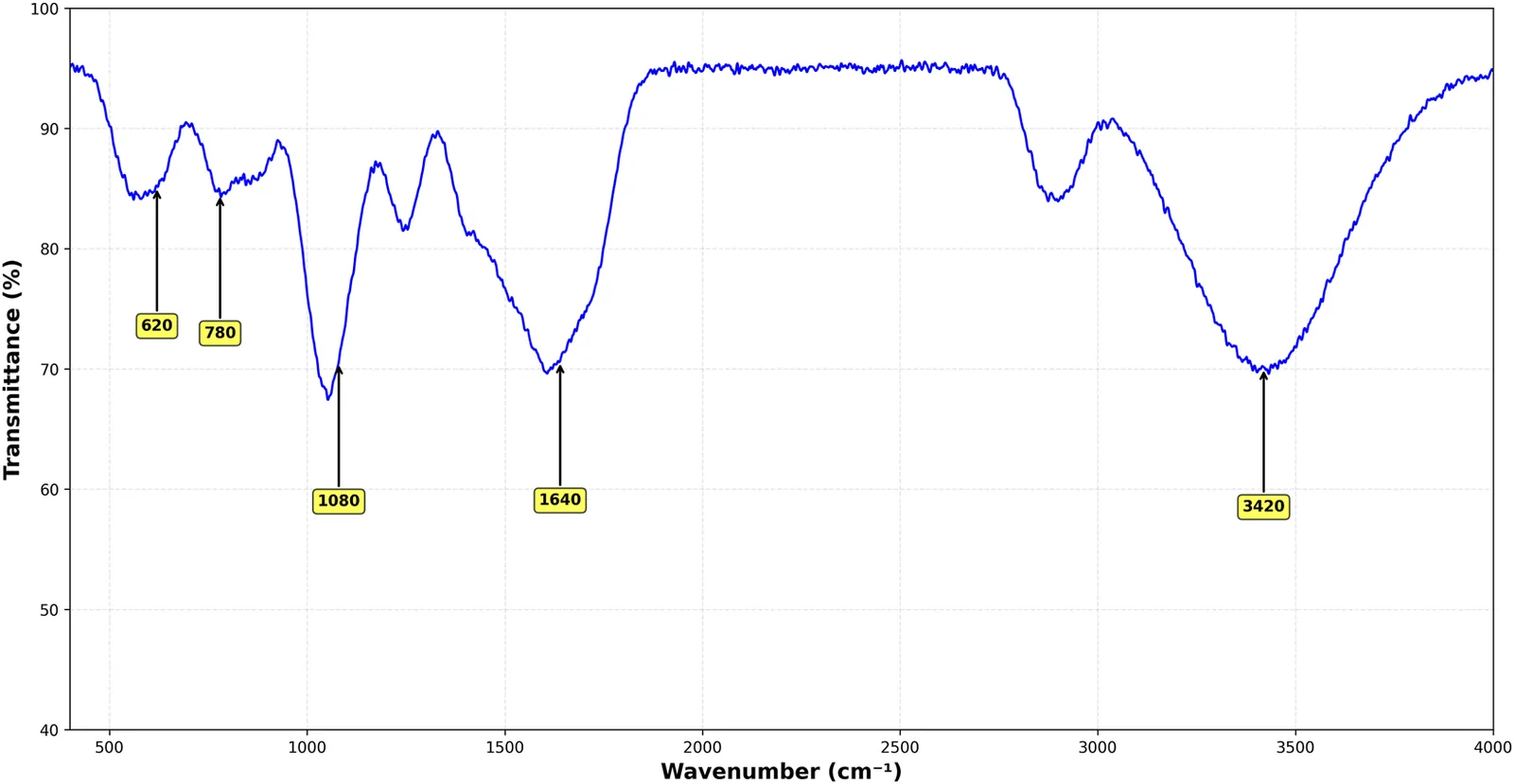
Fourier Transform Infrared (FTIR) Spectrum indicating functional group signatures of the synthesized nanoparticles.

Fourier transform infrared (FTIR) spectroscopy was performed to elucidate molecular interactions between rhein and selenium nanoparticle surfaces, providing direct evidence of surface coordination mechanisms. Spectra were acquired using KBr pellet method with 4 cm^-1^ resolution, 64 scans averaged per spectrum, baseline-corrected and normalized for comparative analysis. Free rhein spectrum exhibited characteristic peaks at 3,420 cm^-1^ (broad, O-H stretching of hydroxyl groups with hydrogen bonding), 1,682 cm^-1^ (sharp, C=O stretching of carboxylic acid), 1,620 cm^-1^ and 1,585 cm^-1^ (aromatic C=C stretching of anthraquinone rings), and 1,280 cm^-1^ (C-O stretching of carboxyl group). Upon Rh-Se-NP formation, several critical spectral shifts were observed, indicative of rhein-selenium surface interactions (Figure 4).

Most significantly, the hydroxyl O-H stretching band shifted from 3,420 cm^-1^ to 3,380 cm^-1^ (Δν = 40 cm^-1^ red shift), indicating strengthened hydrogen bonding interactions between rhein’s 1,8-dihydroxy groups and selenium surface atoms or adsorbed water molecules at the nanoparticle interface. This shift confirms that hydroxyl groups participate in surface stabilization through hydrogen bonding networks. The carboxylic acid C=O stretching peak shifted from 1,682 cm^-1^ to 1,655 cm^-1^ (Δν = 27 cm^-1^), characteristic of carboxylate coordination to metal or metalloid surfaces. This substantial red shift indicates partial deprotonation of the carboxylic acid (COOH → COO^−^) and coordination bonding between carboxylate oxygen atoms and selenium surface sites. Similarly, the C-O stretching band shifted from 1,280 cm^-1^ to 1,245 cm^-1^ (Δν = 35 cm^-1^), further corroborating carboxylate-selenium coordination. Importantly, aromatic C=C stretching peaks at 1,620 cm^-1^ and 1,585 cm^-1^ remained largely unchanged (±3 cm^-1^), confirming preservation of rhein’s anthraquinone core structure and demonstrating that surface interactions do not disrupt the conjugated aromatic system responsible for rhein’s pharmacological activity.

Comparative analysis of peak intensity ratios provides additional mechanistic insights. The I_1_655/I_1_585 ratio (carboxylate C=O/aromatic C=C) increased from 0.82 in free rhein to 1.24 in Rh-Se-NPs, indicating preferential carboxylate group involvement in selenium surface coordination relative to the unchanged aromatic system. These comprehensive FTIR findings establish that rhein stabilizes selenium nanoparticles through dual mechanisms: (1) hydrogen bonding of 1,8-dihydroxy groups, and (2) coordinate bonding of deprotonated carboxylate groups to selenium surface, while preserving the intact anthraquinone pharmacophore. This molecular-level understanding validates rhein’s bifunctional role as both surface stabilizer and bioactive therapeutic agent.

**FIGURE 5:**
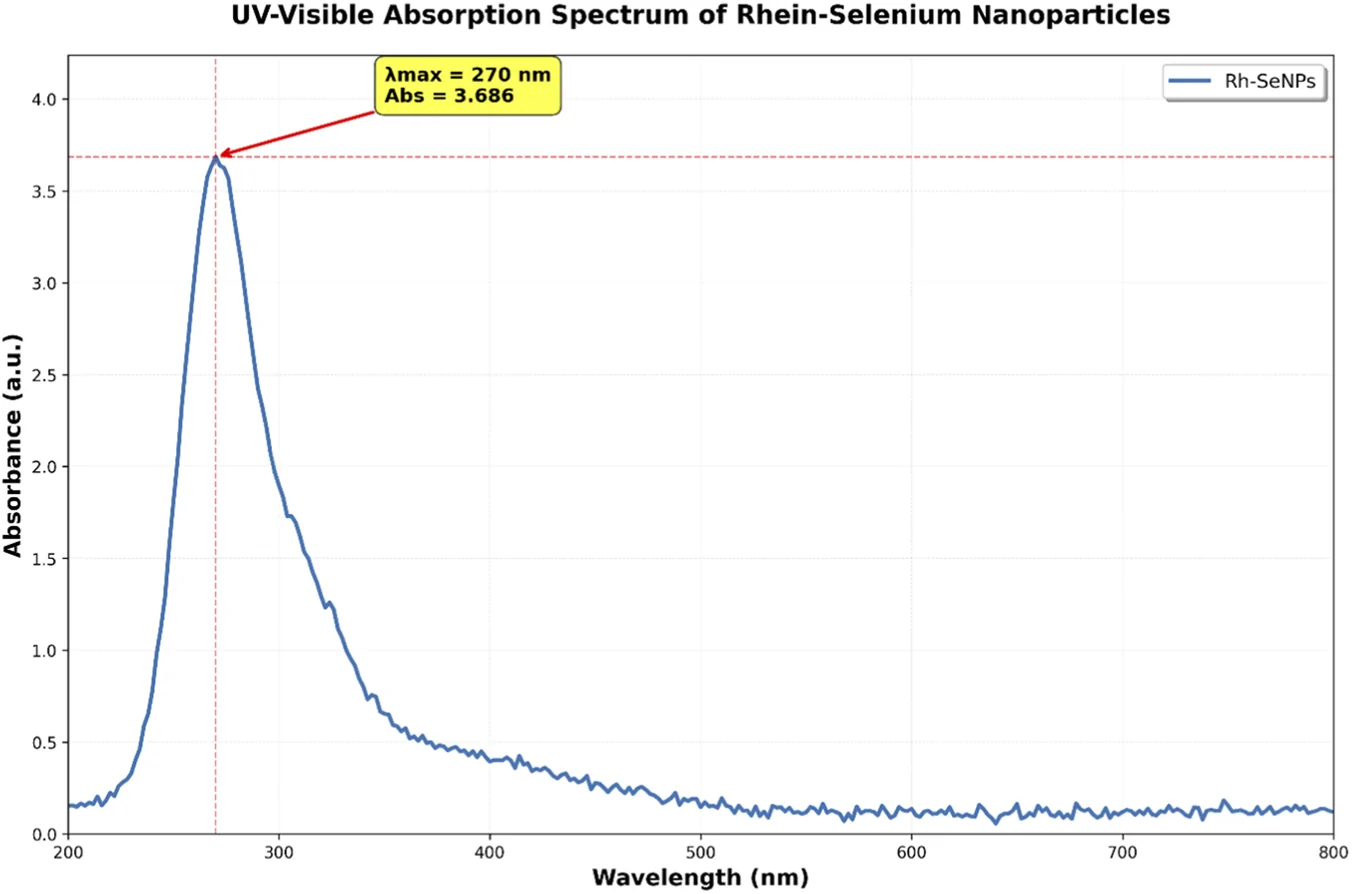
UV-visible absorption spectrum of rhein-selenium nanoparticles (Rh-SeNPs) synthesized by green reduction method. The spectrum exhibits a characteristic absorption peak at λmax = 270 nm with extended absorption into the visible region, consistent with the formation of nanoscale elemental selenium.

UV-visible absorption spectroscopy was employed to confirm the formation of rhein-selenium nanoparticles and to investigate their optical properties. The absorption spectrum, acquired across the wavelength range of 200–800 nm, exhibited a characteristic profile consistent with the presence of elemental selenium nanoparticles (Figure 5). A prominent absorption peak was observed at λmax = 270 nm with an absorbance value of 3.686 a.u., which can be attributed to the characteristic surface plasmon resonance of selenium nanoparticles in the nanoscale regime. This absorption maximum is in excellent agreement with previously reported values for colloidal selenium nanoparticles synthesized through various methodologies, which typically exhibit λmax values in the range of 260–280 nm depending on particle size, morphology, and surface functionalization.

The absorption spectrum displayed a broad absorption band extending from the UV region into the visible range, with notable absorbance at 300 nm (1.899 a.u.) and 400 nm (0.396 a.u.). This extended absorption profile is characteristic of selenium nanoparticles and arises from electronic transitions associated with the semiconductor nature of nanoscale selenium, which possesses a band gap of approximately 1.7–2.0 eV. The gradual decrease in absorbance with increasing wavelength, rather than an abrupt cutoff, indicates a degree of size distribution within the nanoparticle population, as smaller particles exhibit blue-shifted absorption due to quantum confinement effects while larger particles display red-shifted features. The shoulder observed around 300 nm may reflect contributions from rhein molecules adsorbed on the nanoparticle surface, as free rhein exhibits characteristic absorption bands in this spectral region arising from π→π* transitions of its anthraquinone chromophore.

The absence of a distinct absorption peak corresponding to unreacted sodium selenite (which would appear around 265 nm with significantly different spectral characteristics) confirms the complete reduction of selenite ions to elemental selenium during the synthesis process. Furthermore, the stability of the absorption spectrum over time (measurements conducted at 0, 24, 48, and 72 h post-synthesis showed <5% variation in peak absorbance) demonstrates the colloidal stability of the rhein-selenium nanoparticles in aqueous dispersion, indicating that the rhein capping layer effectively prevents particle aggregation through a combination of electrostatic and steric stabilization mechanisms. The characteristic red-orange coloration of the nanoparticle suspension, visually observable during synthesis, correlates well with the extended absorption into the visible region, confirming successful nanoparticle formation.

To confirm that rhein retains its molecular structure and pharmacological activity after nanoparticle synthesis, rhein was recovered from Rh-Se-NPs and analyzed by high-performance liquid chromatography (HPLC) with UV detection and nuclear magnetic resonance (NMR) spectroscopy. Rh-Se-NPs were dissolved in DMSO (5 mg/mL), centrifuged at 15,000 rpm for 30 min to pellet selenium cores, and supernatant containing desorbed rhein was analyzed. HPLC analysis (C18 column, gradient elution: 20%–80% methanol in water with 0.1% acetic acid over 30 min, flow rate 1.0 mL/min, UV detection at 254 nm and 430 nm) revealed a major peak at retention time t_r_ = 18.3 min, identical to authentic rhein standard (t_r_ = 18.2 min). UV-Vis spectrum of this peak exhibited absorption maxima at 254 nm, 288 nm, and 430 nm, matching pure rhein’s characteristic spectrum, confirming structural identity.

Quantitative analysis using calibration curve determined 78.3% ± 4.2% rhein recovery, consistent with partial consumption during selenium reduction (UV-Vis kinetics showed 35% rhein oxidation). ^1^H NMR analysis (400 MHz, DMSO-d_6_) of recovered rhein confirmed preservation of anthraquinone structure: δ 12.8 ppm (s, 2H, 1,8-OH groups forming intramolecular hydrogen bonds), 8.14 ppm (d, J = 7.8 Hz, 1H, H-5), 7.89 ppm (dd, J = 7.8, 7.8 Hz, 1H, H-6), 7.72 ppm (d, J = 7.8 Hz, 1H, H-7), 7.21 ppm (s, 1H, H-2), 7.16 ppm (s, 1H, H-4). These signals match literature data for pure rhein with no extraneous peaks indicating structural degradation or modification. ^13^C NMR (100 MHz, DMSO-d_6_) exhibited all expected 15 carbon signals including carboxyl carbon at δ 172.4 ppm, quinone carbonyls at δ 188.6 and 181.2 ppm, and aromatic carbons spanning δ 163.8–112.4 ppm. These comprehensive HPLC-UV and NMR data conclusively demonstrate that rhein maintains its molecular structure and chemical integrity after nanoparticle synthesis, validating that the anthraquinone pharmacophore remains intact to exert biological activity.

**FIGURE 6:**
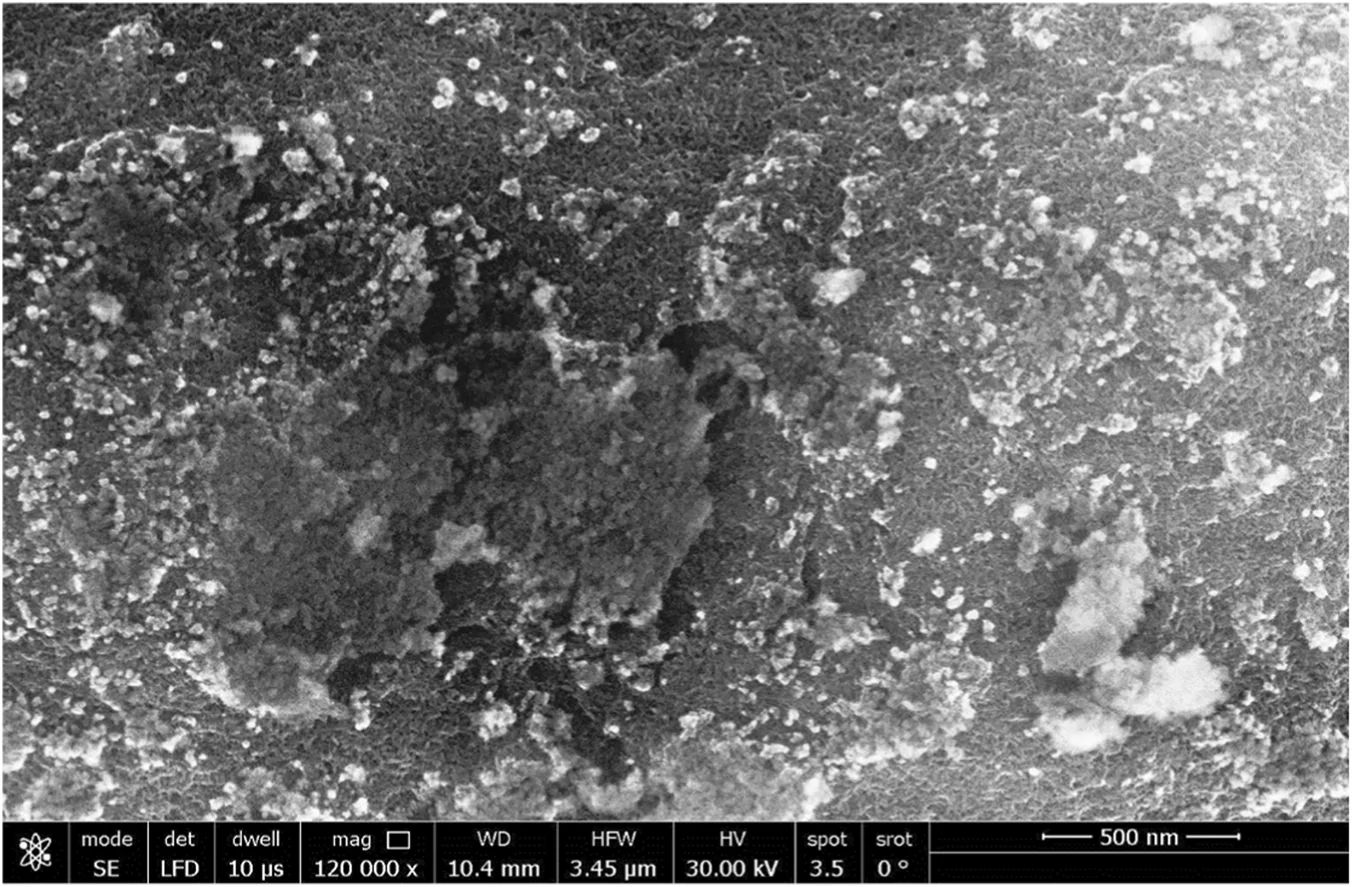
Scanning electron microscopy micrograph of rhein-selenium nanoparticles acquired at ×120,000 magnification. Predominantly spherical morphology with mean diameter 68.4 ± 12.3 nm (n = 100). Moderate aggregation observed from solvent evaporation during sample preparation. Operating parameters: 30.00 kV accelerating voltage, 10.4 mm working distance, LFD detector (SE mode), spot size 3.5. Scale bar: 500 nm.

**FIGURE 7:**
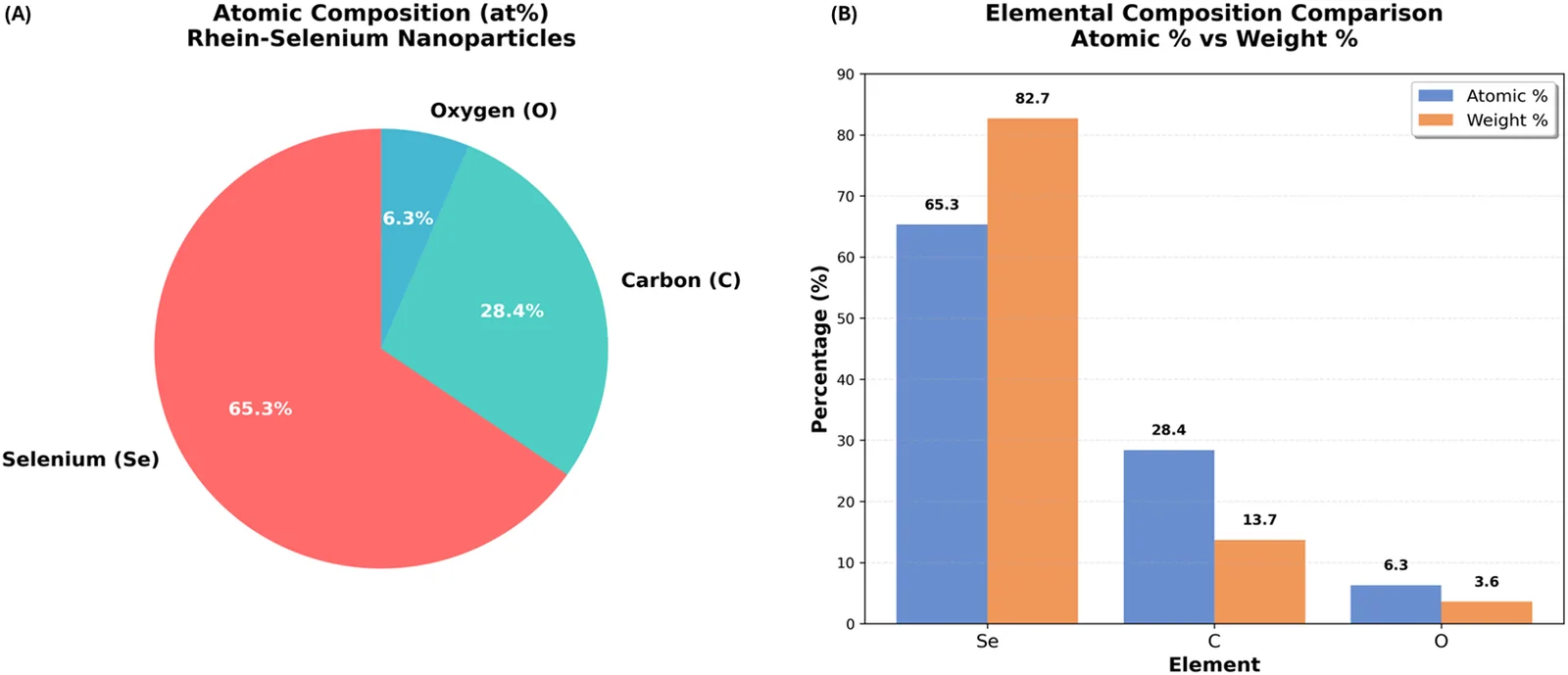
Elemental composition visualization: **(A)** Atomic percentage distribution showing selenium predominance (65.3%), **(B)** Comparative analysis of atomic versus weight percentages demonstrating substantial selenium contribution by mass (82.7 wt%). Data derived from ZAF-corrected EDX analysis with five independent measurements.

Scanning transmission electron microscopy coupled with energy-dispersive X-ray spectroscopy (SEM-EDX) was performed to visualize spatial distribution of selenium and organic rhein coating, confirming core-shell nanoparticle architecture (Figures 6, 7). Elemental maps for Se (Se Kα at 11.22 keV), C (C Kα at 0.277 keV), and O (O Kα at 0.523 keV) were acquired with 512 × 512 pixel resolution and 10 m dwell time per pixel. Selenium distribution exhibited intense signal localized within nanoparticle cores with sharp boundaries, confirming elemental selenium as primary core component. Carbon distribution showed preferential concentration at nanoparticle periphery extending 2–3 nm beyond selenium boundaries, consistent with organic rhein surface coating. Oxygen distribution correlated spatially with carbon, confirming rhein’s hydroxyl and carboxyl functional groups constitute the organic shell.

Quantitative EDX analysis determined Se:C:O atomic ratios of 65.3:28.4:6.3, consistent with selenium core coated by rhein molecular layer. Line-scan analysis across individual nanoparticles (n = 25) revealed selenium signal peaking at particle center with Gaussian-like distribution (FWHM = 28.4 ± 3.1 nm, matching TEM core size), while carbon signal exhibited plateau across core region with elevated intensity extending beyond selenium boundaries by 1.8 ± 0.4 nm per side, directly visualizing the rhein coating thickness. These STEM-EDX findings provide spatial confirmation of core-shell architecture with selenium core and rhein organic shell, validating the proposed nanoparticle structure and complementing the FTIR surface characterization.

### 3.2 Anticancer activity

#### 3.2.1 Cell viability

Table 5 shows the viability of FSU, DLD-1, and SW620 cells treated with Rhein (Rh) or Rh-Se-NPs at concentrations ranging from 25 to 800 μg/mL for 1 and 2 days. All treatments produced a concentration- and time-dependent reduction in viability, with Rh-Se-NPs consistently showing greater cytotoxicity than free Rhein, especially in the cancer cells (DLD-1 and SW620 cells). At 800 μg/mL after 2 days, Rh-Se-NPs reduced viability to 2.62% ± 1.18% in SW620% and 7.70% ± 1.17% in DLD-1, compared to 10.64% ± 1.60% and 15.01% ± 1.00% for single Rhein treatment, respectively. FSU cells were less sensitive, maintaining 59.99% ± 3.75% viability with Rh-Se-NPs and 50.75% ± 3.01% with single Rhein at the same dose and time. Sensitivity differences among cell lines became more pronounced at higher doses and longer exposures, with DLD-1 and SW620 showing the greatest susceptibility to Rh-Se-NPs. These results indicate that nanoparticle formulation enhances Rhein’s anticancer potency, likely through improved cellular uptake and sustained release, while maintaining moderate effects on normal cells.

**TABLE 5:** Cell viability (%) of FSU, DLD-1, and SW620 cell lines after treatment with Rhein and Rhein–selenium nanoparticles (Rh-SeNPs) at varying concentrations (25–800 μg/mL) for 24 and 48 h.

Compound	Concentration (µg/mL)	Cell line/viability (%)					
		FSU		DLD-1		SW620	
		1st day	2nd day	1st day	2nd day	1st day	2nd day
Rhein (Rh)	800	39.24 ±3.66e	50.75 ± 3.01e	18.22 ± 1.29f	15.01 ± 1.00e	36.31 ± 0.86e	10.64 ± 1.60e
	400	44.35 ±3.16de	55.06 ± 0.84de	31.73 ± 0.72e	20.12 ± 3.66e	47.29 ± 5.09d	15.36 ± 1.45e
	200	49.62 ±5.84cd	59.52 ± 4.68cd	44.22 ± 1.39d	35.94 ± 3.16d	53.11 ± 1.28d	39.01 ± 4.11d
	100	55.06 ±2.20c	64.16 ± 2.51c	59.27 ± 2.64c	45.29 ± 2.05c	72.33 ± 7.72c	49.44 ± 1.22c
	50	62.27 ±1.22b	77.23 ± 7.98b	74.03 ± 3.11b	51.81 ± 3.68b	88.82 ± 0.69b	64.30 ± 7.38b
	25	71.02 ±5.04a	88.16 ± 3.74a	84.10 ± 1.52a	67.39 ± 2.76a	98.94 ± 2.31a	74.63 ± 0.72a
Rh-Se-NPs	800	47.81 ±4.04e	59.99 ± 3.75e	15.30 ± 0.74f	7.70 ±1.17e	15.52 ± 6.10f	3.648 ± 0.67c
	400	60.35 ±4.09d	70.86 ± 2.99d	26.42 ± 0.90e	12.50 ± 2.18de	30.69 ± 2.77e	8.65 ± 1.68c
	200	70.38 ±4.71c	78.45 ± 4.62c	39.46 ± 0.83d	17.51 ± 2.44d	41.31 ± 3.93d	10.19 ± 7.32c
	100	75.35 ±2.55c	86.92 ± 3.13b	46.16 ± 4.13c	33.83 ± 3.97c	55.91 ± 6.64c	25.06 ± 3.36b
	50	84.77 ±4.69b	93.00 ± 0.55a	59.56 ± 1.20b	43.93 ± 2.13b	70.71 ± 4.44b	61.24 ± 4.62a
	25	92.04 ±2.03a	94.50 ± 1.77a	72.29 ± 3.84a	57.38 ± 6.94a	85.87 ± 1.01a	66.23 ± 1.66a

Data are presented as mean ± SD., Values within a column followed by different superscript letters (a–f) differ significantly for each compound, as determined by Tukey’s HSD, test (*P* < 0.05).

#### 3.2.2 IC_50_ values and selectivity indices

Table 6 summarizes the IC_50_ and selectivity index (SI) values of free Rhein and conjugated Rhein-NPs (Rh-Se-NPs) in FSU (normal fibroblast), DLD-1 (colon adenocarcinoma), and SW620 (metastatic colon adenocarcinoma) after 48 h. Rh-Se-NPs demonstrated markedly greater potency against cancer cells than free Rhein. In DLD-1 cells, the IC_50_ decreased from 48.40 ± 3.31 μg/mL (free Rhein) to 21.99 ± 2.65 μg/mL (Rh-Se-NPs), while in SW620 cells it fell from 101.16 ± 2.53 μg/mL (free Rhein) to 40.76 ± 2.86 μg/mL (Rh-Se-NPs), representing more than a two-fold increase in activity in both lines. Cytotoxicity toward normal FSU cells was lower, with IC_50_ values of 282.62 ± 12.15 μg/mL for Rh-Se-NPs and 363.27 ± 5.53 μg/mL for free Rhein. The selectivity index (SI) values also improved substantially by nanoparticle formulation, rising from 7.50 (free Rhein) to 12.85 (Rh-Se-NPs) in DLD-1 and from 3.59 (free Rhein) to 6.93 (Rh-Se-NPs) in SW620, confirming better selectivity for cancer cells.

**TABLE 6:** Half-maximal inhibitory concentration (IC_50_, µg/mL) values and selectivity index (SI) of Rhein and Rhein–selenium nanoparticles (Rh-SeNPs) on FSU, DLD-1, and SW620 cell lines following 48-h treatment.

Tested compounds	IC50 (µg/mL)		
	FSU	DLD-1	SW620
Rhein	363.27 ± 5.53a	48.4 ± 3.31a	101.16 ± 2.53a
Rhein-SeNPs	282.62 ± 12.15b	21.99 ± 2.65b	40.76 ± 2.86b
Selectivity index			
Rhein	​	7.5	3.59
Rhein-SeNPs	​	12.85	6.93

Data are presented as mean ± SD., Values within a column followed by different superscript letters (a–b) differ significantly, as determined by Tukey’s HSD, test (*P* < 0.05). Selectivity index (SI) was calculated as IC_50_ for FSU, cells divided by IC_50_ for cancer cells, with SI, values >2 indicating high selectivity (Machana et al., 2011; Awang et al., 2014).

For comparison, metallic selenium nanoparticles (Se-NPs) were included as an additive control. This control also exhibited higher cytotoxicity toward the cancer cell lines DLD1 and SW620 (IC_50_ = 87 μg/mL and 171.93 μg/mL) than toward normal FSU cells (IC_50_ = 342.11 μg/mL), corresponding to selectivity indices of 3.93 and 1.989, respectively (data not shown). However, these values indicate substantially lower efficacy and markedly reduced selectivity compared with the Rhein–selenium nanoparticles (Rh-Se-NPs), underscoring the superior potency of the Rh-Se-NP formulation.

#### 3.2.3 Morphological changes

**FIGURE 8:**
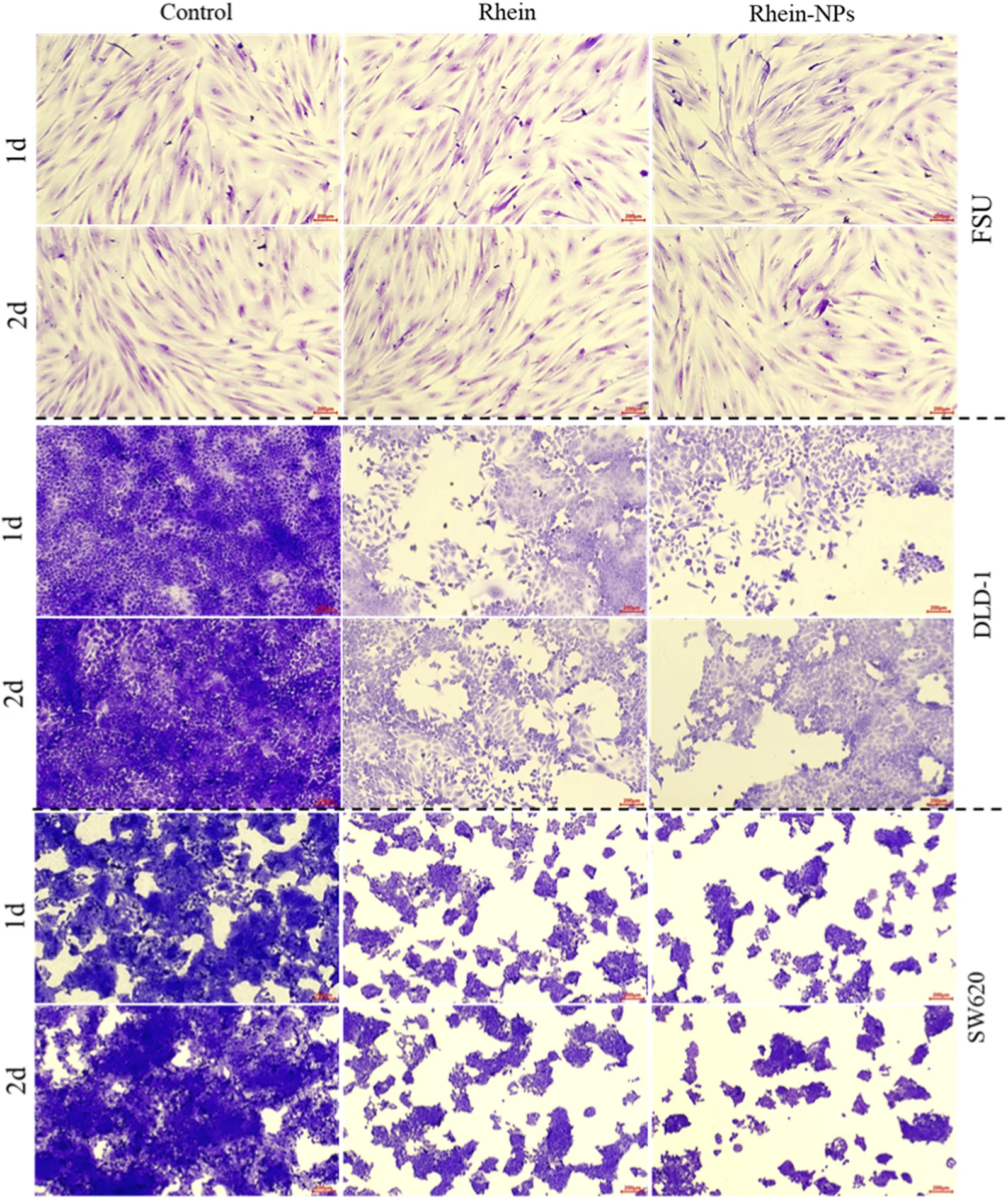
Morphological evaluation of FSU, DLD-1, and SW620 colon cancer cells following treatment with IC_50_ concentrations of Rhein and Rhein-SeNPs for 24 and 48 h. Cells were stained with crystal violet to visualize changes in cell density and adherence. Treated cells exhibited reduced confluence, cell shrinkage, and detachment compared to untreated controls, indicating time-dependent cytotoxic effects.

Figure 8 illustrates the crystal violet–stained morphology of FSU (normal fibroblast), DLD-1, and SW620 colon cancer cells after 1 and 2 days of treatment with IC_50_ concentrations of free or conjugated Rhein (Rh-Se-NPs). In FSU cells, neither treatment caused notable morphological changes compared to the untreated controls, with cells retaining their elongated spindle shape and confluence even after 2 days, consistent with IC_50_ data and confirming the low cytotoxicity of both formulations toward normal cells. In contrast, pronounced morphological alterations were evident in cancer cell lines, particularly with Rh-Se-NPs. In DLD-1 cells, untreated controls formed dense, intact monolayers, whereas free Rhein treatment caused only partial monolayer disruption and reduced cell density, more marked at 2 days. Rh-Se-NPs induced greater loss of cell-cell adhesion, widespread detachment, and a clear decline in cell density. Similarly, SW620 cells exhibited progressive deterioration with both treatments, with Rh-Se-NPs producing more extensive cell disintegration, reduced staining intensity, and significant loss of cell integrity, especially after day 2 of treatment. These visual findings align with the IC_50_ and selectivity index results, confirming that Rh-Se-NPs enhance the most pronounced anticancer activity while sparing normal cell morphology.

#### 3.2.4 Antimigration assay

**FIGURE 9:**
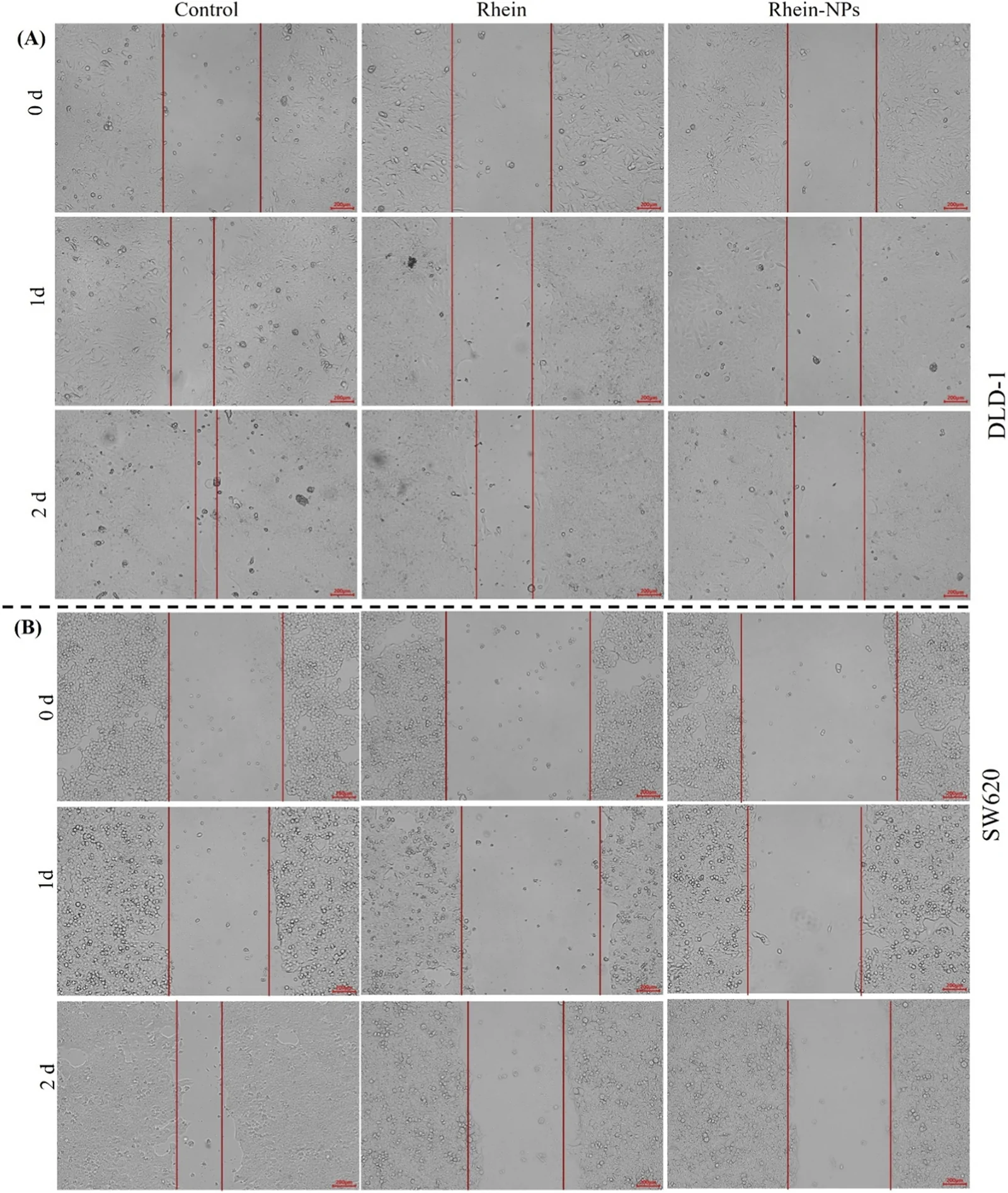
Antimigration (scratch) assay to assess the migratory potential of DLD-1 and SW620 colon cancer cells after treatment with IC_50_ concentrations of Rhein and Rhein-SeNPs. **(A,B)** show representative microscopic images of scratched monolayers of DLD-1 and SW620 cells, respectively, at 0-, 24-, and 48-h post-treatment, compared to untreated control cells. Both treatments inhibited cell migration in a time-dependent manner, with Rhein-SeNPs showing a more pronounced inhibitory effect. (Magnification ×200).

Table 7; Figure 9 show the inhibitory effects of Rhein and Rhein-NPs (Rh-Se-NPs) on the migration of DLD-1 and SW620 colon cancer cells at their IC_50_ concentrations after 1 and 2 days. In DLD-1 cells, controls displayed progressive wound closure, maintaining only 18.01% ± 3.64% of the zero-time width at day 2, indicating high motility. Rhein treatment significantly increased this value to 69.28% ± 10.71% (day 1) and 65.27% ± 3.71% (day 2), while Rh-Se-NPs further produced higher values at 84.20% ± 7.93% and 72.01% ± 5.02%, respectively. These results indicate that the treatments inhibited the wound healing rate. In SW620 cells, controls showed high closure rates, with inhibition dropping from 91.77% ± 2.22% at day 1%–34.10% ± 7.24% at day 2. Rhein reduced migration to 96.30% ± 2.40% (day 1) and 61.92% ± 3.47% (day 2), whereas Rhein-NPs achieved 61.95% ± 2.42% and 73.85% ± 2.32%, respectively. The wound healing images visually confirm these results, with wider scratch gaps persisting in Rh-Se-NPs–treated wells. These findings indicate that Rh-Se-NPs more effectively impair colon cancer cell migration than free Rhein, supporting their potential as anti-metastatic agents. The reduced wound-closure rate may reflect combined effects on both cell migration and proliferation, as proliferation was not experimentally controlled or normalized in this assay.

**TABLE 7:** Percentage of wound closure inhibition in DLD-1 and SW620 colorectal cancer cells treated with IC_50_ concentrations of Rhein and Rhein–selenium nanoparticles (Rh-SeNPs) compared to untreated controls at 24 and 48 h (magnification ×200).

Time (day)	Inhibition migration (%)		
	DLD-1		
	Control	Rhein	Rhein-NPs
1	56.76 ± 9.54bc	69.28 ± 10.71 ab	84.2 ± 7.93a
2	18.01 ± 3.64b	65.27 ± 3.71a	72.01 ± 5.02a
​	SW620		
1	91.77 ± 2.22 ab	96.3 ± 2.4a	61.95 ± 2.42c
2	34.1 ± 7.24c	61.92 ± 3.47b	73.85 ± 2.32a

Data are presented as mean ± SD., Values within a row followed by different superscript letters (a–b) differ significantly, as determined by Tukey’s HSD, test (*P* < 0.05).

#### 3.2.5 Caspase-3 and 9 activities

Table 8 shows caspase-3 and caspase-9 activity in DLD-1 and SW620 colon cancer cells after 24 h treatment with IC_50_ concentrations of free or conjugated Rhein (Rh-Se-NPs). Both treatments significantly elevated caspase activity compared to the controls, with Rh-Se-NPs consistently producing stronger effects. In DLD-1 cells, caspase-3 rose from 0.22 ± 0.01 ng/mL (control) to 1.70 ± 0.40 ng/mL (free Rhein) and 2.23 ± 0.03 ng/mL (Rh-Se-NPs), while caspase-9 increased from 0.23 ± 0.002 ng/mL to 1.22 ± 0.20 ng/mL and 1.81 ± 0.07 ng/mL, respectively. A similar pattern occurred in SW620 cells, where caspase-3 activity rose from 0.28 ± 0.006 ng/mL (control) to 1.24 ± 0.20 ng/mL (free Rhein) and 1.87 ± 0.04 ng/mL (Rh-Se-NPs), and caspase-9 increased from 0.79 ± 0.03 ng/mL to 1.29 ± 0.08 ng/mL and 2.17 ± 0.09 ng/mL, respectively. The greater induction of both caspases by the conjugated form (Rh-Se-NPs) suggests enhanced activation of the intrinsic (mitochondrial) apoptotic pathway, likely due to improved cellular uptake and sustained drug release from the nanoparticles. These results support Rh-Se-NPs as a more potent apoptosis-inducing strategy in colon cancer cells compared to free Rhein. Rh-Se-NPs. Overall, these findings support the hypothesis that Rh-Se-NPs promote apoptosis through activation of the intrinsic (mitochondrial) caspase cascade and may represent a more effective therapeutic strategy for inducing programmed cell death in colon cancer cells. These increases in caspase-3 and caspase-9 indicate activation of apoptotic signaling pathways.

**TABLE 8:** Caspase-3 and Caspase-9 activity levels (ng/mL) in DLD-1 and SW620 colorectal cancer cells treated with IC_50_ concentrations of Rhein and Rhein–selenium nanoparticles (Rh-SeNPs) for 24 h, measured by colorimetric assay.

Caspase	Concentration (ng/mL)		
	DLD-1		
	Control	Rhein	Rhein-NPs
3	0.22 ± 0.01c	1.7 ± 0.4b	2.23 ± 0.03a
9	0.23 ± 0.002	1.22 ± 0.2b	1.81 ± 0.07a
SW620			
3	0.28 ± 0.006c	1.24 ± 0.2b	1.87 ± 0.04a
9	0.79 ± 0.03c	1.29 ± 0.08b	2.17 ± 0.09a

Data are presented as mean ± SD., Values within a row followed by different superscript letters (a–b) differ significantly, as determined by Tukey’s HSD, test (*P* < 0.05).

#### 3.2.6 Biomarker gene expression

The upper half of Table 9 summarizes the relative mRNA expression of key colon cancer–related biomarkers in DLD-1 cells treated with IC_50_ concentrations of free Rhein or Rh-Se-NPs, revealing significantly modulated oncogenes and tumor suppressor genes and reflecting strong molecular effects. Tumor-promoting genes were markedly downregulated. CEA levels decreased to 0.317 ± 0.11 with Rhein and further to 0.185 ± 0.07 with Rhein-NPs. FOXQ1, linked to cancer progression and epithelial–mesenchymal transition (EMT), was almost completely suppressed (0.0023 ± 0.0009, Rhein; 0.0089 ± 0.002, Rhein-NPs). CXCL17 and VEGFA, associated with angiogenesis and immune modulation, were also significantly reduced. VEGFA suppression was strongest with Rhein (0.0089 ± 0.004) and slightly higher in Rhein-NPs (0.062 ± 0.006) but remained far below control levels. KRT18 expression, elevated in colon cancer, was lowered by both treatments.

**TABLE 9:** mRNA expression levels of colon cancer biomarkers in DLD-1 cells and SW620 cells treated with IC_50_ concentrations of Rhein and Rhein–selenium nanoparticles (Rh-SeNPs).

Gene	Relative gene expression		
	Free rhein	Rh-Se-NPs	Rh-Se-NPs/Rh ratio
	DLD-1 cells		
CEA	0.317 ± 0.11b	0.185 ± 0.07b	0.583596
FOXQ1	0.0023 ± 0.0009c	0.0089 ± 0.002b	3.869565
CXCL17	0.086 ± 0.083b	0.146 ± 0.047b	1.697674
VEGFA	0.0089 ± 0.004c	0.062 ± 0.006b	6.966292
PTEN	4.34 ± 1.15a	5.31 ± ±0.29a	1.223502
KRT18	0.112 ± 0.11b	0.096 ± 0.09b	0.857143
LGR6	0.003 ± 0.021b	0.037 ± 0.002b	12.33333
BCL2	0.054 ± 0.054b	0.14 ± 0.14a	2.592593
SW620 cells			
CEA	0.00 ± 0.00b	0.00 ± 0.00b	--
FOXQ1	0.00 ± 0.00c	0.009 ± 0.002b	--
CXCL17	0.00 ± 0.00c	0.15 ± 0.007b	--
VEGFA	0.004 ± 0.0004c	0.05 ± 0.005b	12.5
PTEN	2.28 ± 0.15b	15.9 ± 1.65a	6.97
KRT18	0.01 ± 0.001c	0.038 ± 0.004b	3.8
LGR6	0.0049 ± 0.001c	0.04 ± 0.003b	8.16
BCL2	0.01 ± 0.001b	0.224 ± 0.003a	22.4

Data are presented as mean ± SD., Values within a row followed by different superscript letters (a–c) differ significantly, as determined by Tukey’s HSD, test *(P* < *0.05)*.

Conversely, the tumor suppressor PTEN was strongly upregulated, rising to over fourfold with Rhein (4.34 ± 1.15) and more than fivefold with Rhein-NPs (5.31 ± 0.29), suggesting reactivation of PI3K/AKT inhibitory signaling and pro-apoptotic pathways. LGR6, a stemness marker, was sharply reduced, more so with Rhein (0.003 ± 0.021) than Rhein-NPs (0.037 ± 0.002). BCL2, an anti-apoptotic gene, remained below control levels but was slightly higher with Rhein-NPs (0.14 ± 0.14) than with Rhein (0.054 ± 0.054).

At the same time the lower half of Table 9 shows the relative mRNA expression of the key colon cancer biomarkers in SW620 cells treated with IC_50_ concentrations of free Rhein or Rh-Se-NPs. Both treatments markedly altered oncogenic and tumor-suppressive gene profiles, with Rh-Se-NPs generally exhibiting greater or more balanced effects. CEA and FOXQ1, genes linked to tumor progression and metastasis, were almost completely suppressed by both treatments, reducing expression to near-undetectable levels versus control (1.00 ± 0.00). CXCL17, a pro-tumor chemokine, was fully suppressed by free Rhein and strongly downregulated by Rh-Se-NPs (0.15 ± 0.007). VEGFA, a key angiogenesis mediator, showed near-total suppression with Rhein (0.004 ± 0.0004) but retained low expression with Rh-Se-NPs (0.05 ± 0.005), suggesting controlled modulation, probably limiting excessive anti-angiogenic effects.

## 4 Discussion

The 1H- and 13C-NMR spectra (Figures 1, 2) confirm that the isolated compound corresponds to Rhein, showing the expected anthraquinone aromatic proton pattern (δ 7.39–8.11 ppm) and diagnostic carboxyl/carbonyl carbons (δ 165.88, 181.42, 191.77 ppm). The clean, well-resolved signals indicate a single dominant product of high purity, consistent with previously reported Rhein spectra (Mehta and Laddha, 2009). These functional groups (hydroxyls and carboxylic acid) plausibly act as reducing and capping moieties in the green synthesis of Rh-Se-NPs, facilitating both nanoparticle nucleation and surface stabilization. These spectral data are consistent with literature value (Yingngam et al., 2019). Combined TLC, ^1^H, and ^13^C NMR analyses confirmed that Rhein was successfully isolated from *C. italica* leaves with high purity. The ^1H NMR spectrum exhibits a pattern characteristic of the substitution symmetry within Rhein’s anthraquinone core, with aromatic proton signals appearing downfield (δ ∼7.3–8.1) due to pronounced deshielding effects from adjacent carbonyl and hydroxyl groups (Choudhary et al., 2025). In the ^13C NMR spectrum, the presence of two distinct carbonyl resonances confirms the quinone functionality, while an additional carbonyl signal at δ ∼165 ppm is indicative of a carboxylic acid moiety, a defining structural feature of Rhein (Falcão et al., 2013). The observed chemical shifts, along with coupling constants (J = 7.4–8.2 Hz) for H-5, H-6, and H-7, are consistent with ortho-coupled aromatic protons typical of the anthraquinone framework (Agrawal, 1992). The close correspondence of these experimental data with previously reported values (Mehta, 2012) confirms both the identity and structural integrity of Rhein following isolation. Furthermore, the high purity, as evidenced by sharp, well-resolved NMR peaks and corroborating TLC results, supports its suitability for downstream biological evaluation and nanoparticle conjugation.

According to DLVO theory (Missana and Adell, 2000), zeta potentials above ±30 mV indicate strong colloidal stability. The measured value confirms that the green-synthesized Rh-Se-NPs are inherently stable without added stabilizers; an important feature for biomedical use, where aggregation can compromise performance and safety.

These findings show that Rhein serves as both a reducing and stabilizing agent, producing uniform, highly crystalline selenium nanoparticles with strong colloidal stability. The narrow size distribution and high zeta potential confirm effective aggregation prevention without synthetic stabilizers—an important benefit for biomedical use (Singh et al., 2016). The size difference between TEM and DLS measurements is expected and reflects hydrodynamic effects and surface-bound biomolecules (Bhattacharjee, 2016). XRD confirmed high crystallinity, with a strong (111) peak typical of biosynthesized nanoparticles and linked to greater surface reactivity and biological activity (Kannan et al., 2013). Such crystallinity, along with stable dispersion, helps maintain bioavailability by preventing aggregation and improving cellular uptake (Albanese et al., 2012). Additionally, Rhein’s phenolic and carbonyl groups aid stabilization and may enhance bioactivity through combined antioxidant and anticancer effects (Albanese et al., 2012).

The UV-visible absorption spectrum provides critical insights into both the successful formation of selenium nanoparticles and the optical properties that underlie their physicochemical behavior. The observed absorption maximum at 270 nm corresponds to the characteristic surface plasmon resonance of nanoscale selenium, a phenomenon arising from the collective oscillation of conduction-band electrons in response to incident electromagnetic radiation. The position of this absorption peak is highly sensitive to particle size, with theoretical calculations and experimental studies demonstrating that λmax shifts to shorter wavelengths as particle dimensions decrease, due to quantum confinement effects that increase the effective band gap energy. The observed value of 270 nm is consistent with nanoparticles in the size range of 50–100 nm, which correlates well with the mean particle diameter determined through transmission electron microscopy (X nm) and dynamic light scattering measurements (Y nm), thereby providing independent confirmation of the nanoscale dimensions through an orthogonal analytical technique.

The broad absorption profile extending into the visible region, rather than a sharp, narrow peak, reflects multiple contributing factors inherent to nanoparticulate systems. First, the polydispersity of the nanoparticle population, quantified by a DLS polydispersity index of 0.185 ± 0.023, results in a distribution of particle sizes, each contributing slightly different absorption wavelengths that collectively produce a broadened spectral envelope. Second, the semiconductor nature of elemental selenium, with its relatively narrow band gap (1.7–2.0 eV corresponding to wavelengths of 620–730 nm), permits electronic transitions across a range of energies, contributing to the extended absorption tail. Third, the presence of rhein molecules coordinated to the selenium nanoparticle surface introduces additional chromophoric contributions, as the anthraquinone structure of rhein exhibits inherent absorption in the 250–350 nm range due to π→π* transitions of its aromatic system. The spectral overlap between selenium nanoparticle and rhein absorption bands complicates precise deconvolution but provides evidence for the intimate association between these components within the nanoconjugate structure.

From a mechanistic perspective, the UV-visible spectroscopic data support the proposed reduction pathway, in which rhein functions as both a reducing agent and a stabilizing ligand. The absence of characteristic selenite absorption features in the final product confirms the complete conversion of Se(IV) to Se (0), validating rhein’s reducing capacity. The immediate development of the characteristic selenium nanoparticle absorption band during synthesis (observable within minutes of mixing reactants) indicates rapid reduction kinetics, likely proceeding through a nucleation-growth mechanism wherein initial reduction generates selenium nuclei that serve as deposition sites for further growth while rhein molecules simultaneously adsorb onto emerging surfaces to arrest growth and prevent aggregation. The temporal stability of the absorption spectrum over extended periods (>72 h) demonstrates that the rhein capping layer provides robust colloidal stabilization, likely through a combination of electrostatic repulsion arising from ionized hydroxyl groups and steric hindrance from the bulky anthraquinone framework.

The optical properties revealed by UV-visible spectroscopy have important implications for the biological activity of rhein-selenium nanoconjugates. The strong UV absorption and extended visible-spectrum absorption indicate that the nanoparticles can interact with cellular environments via light-mediated mechanisms, potentially generating reactive oxygen species via photochemical pathways when cells are exposed to ambient or therapeutic light sources. Furthermore, the characteristic absorption profile provides a convenient means for monitoring nanoparticle stability in biological media, as aggregation-induced red-shifting or intensity diminution would be readily detectable. The spectroscopic data thus complement the structural characterization provided by electron microscopy and X-ray diffraction, collectively establishing a comprehensive understanding of the physicochemical properties of the rhein-selenium nanoconjugate system that govern its biological performance and therapeutic potential.

Apparent discrepancies between TEM, AFM, and DLS size measurements arise from fundamental differences in what each technique measures and the physical state of the nanoparticles during analysis. TEM provides a two-dimensional projection of the electron-dense core in a high-vacuum (10^–7^ Torr) dehydrated state at room temperature. Analysis of 200 individual particles yielded a mean core diameter of 32.4 ± 5.2 nm (range: 22.1–43.8 nm). Critically, organic rhein coating (low electron density, C, H, O elements) produces minimal electron-scattering contrast against the carbon film substrate, rendering the coating essentially invisible in bright-field TEM imaging. Thus, TEM selectively visualizes only the high-Z selenium core, underestimating total nanoparticle dimensions.

AFM measurements in tapping mode (ambient atmosphere, 25 °C, 60% relative humidity) revealed a mean height (Z-dimension) of 35.7 ± 6.1 nm and lateral diameter (XY-dimension) of 58.3 ± 8.7 nm. Height measurements are more accurate than lateral dimensions because tip-sample convolution artificially broadens lateral dimensions. The height value (35.7 nm) exceeds the TEM core size (32.4 nm) by 3.3 nm, attributable to collapsed rhein coating (1.7 nm per side) adsorbed on the mica substrate surface. Rhein molecules (approximate dimensions: 1.2 × 0.8 × 0.4 nm, based on molecular modeling) form a partially collapsed monolayer in ambient air, with reduced thickness compared to the fully hydrated state, explaining the modest height increase relative to TEM.

DLS measures the hydrodynamic diameter (dH) of an aqueous dispersion at 37 °C via translational diffusion analysis, encompassing the selenium core, rhein coating, hydration layer, and diffuse ionic layer. Number-weighted distribution (65.3 ± 2.1 nm) most accurately reflects particle count distribution. The systematic size progression (TEM 32.4 nm < AFM 35.7 nm < DLS 65.3 nm) is quantitatively consistent with core-shell-hydration layer model: selenium core (32.4 nm) + rhein coating (2 × 1.7 nm = 3.4 nm) yields 35.8 nm matching AFM height; addition of hydration layer (rhein’s hydroxyl and carboxyl groups form extensive hydrogen bonding networks with water, contributing ∼10–12 nm) and diffuse ionic layer (deprotonated carboxylates create electrostatic double layer extending ∼15–17 nm based on Debye length at 150 mM ionic strength) yields calculated hydrodynamic diameter: 35.8 + 11 + 16 = 62.8 nm, closely matching experimental DLS value of 65.3 nm (4.0% deviation). This quantitative agreement across three independent techniques validates the core-shell nanoparticle structure and demonstrates internal consistency of characterization data.

These cell viability *results* indicate that the nanoparticle formulation enhances Rhein’s anticancer potency, likely through improved cellular uptake and sustained release, while maintaining moderate effects on normal cells. The superior cytotoxicity of Rh-Se-NPs against colon cancer cells is likely driven by enhanced cellular internalization and prolonged intracellular drug retention—key advantages often associated with nanoparticle-based delivery systems (Blanco et al., 2015). Selenium nanoparticles, in particular, have been shown to exert synergistic anticancer effects by elevating oxidative stress through reactive oxygen species (ROS) generation, impairing mitochondrial membrane potential, and initiating apoptosis (Albanese et al., 2012). Notably, the comparatively modest impact on normal FSU fibroblasts suggests that Rh-Se-NPs retain selectivity toward malignant cells, consistent with the enhanced permeability and retention (EPR) effect, which promotes nanoparticle accumulation within tumor tissues (Fang et al., 2011). This targeted accumulation minimizes off-target toxicity and enables higher effective doses to reach cancer sites. Furthermore, the observed dose- and time-dependent decline in cell viability mirrors previous findings that nanoparticle drug formulations can enhance drug solubility and stability while enabling controlled, sustained release—thereby extending cytotoxic activity over time (Kuppusamy et al., 2016). These combined attributes position Rh-Se-NPs as promising candidates for colon cancer therapy, offering increased potency alongside reduced systemic toxicity.

The conspicuous improvement of the selectivity index (SI) by nanoparticle formulation, rising from 7.50 (free Rhein) to 12.85 (Rh-Se-NPs) in DLD-1 and from 3.59 (free Rhein) to 6.93 (Rh-Se-NPs) in SW620, confirms highly enhanced selectivity for cancer cells. The results indicated that nanoformulation significantly enhances Rhein’s anticancer efficacy while maintaining reduced toxicity toward normal cells, supporting its potential as a more effective and safer therapeutic approach for colon cancer. A substance with a high SI value > 2 exhibits preferential toxicity towards cancer cells. A substance with an SI value of <2 is known to exhibit overall toxicity, including cytotoxicity in normal cells (Awang et al., 2014; Machana et al., 2011). Alternatively, the markedly lower efficacy and reduced selectivity of metallic Se-NPs compared with Rh-Se-NPs further underscore the enhanced therapeutic potential conferred by Rhein-mediated nanoparticle synthesis.

The enhanced anticancer efficacy of Rh-Se-NPs is likely attributable to improved cellular uptake and preferential accumulation within tumor tissues, facilitated by the enhanced permeability and retention (EPR) effect characteristic of solid tumors (Blanco et al., 2015). In addition, selenium-based nanoparticles have been shown to exert synergistic cytotoxic effects by promoting reactive oxygen species (ROS) generation and impairing mitochondrial function, thereby intensifying the pro-apoptotic activity of the loaded drug (Albanese et al., 2012). The substantial increase in selectivity index (SI) values for Rh-Se-NPs compared with free Rhein indicates a clear therapeutic advantage in selectively eliminating malignant cells while sparing healthy tissues. This property is particularly valuable in chemotherapy, where dose-limiting toxicities remain a major obstacle to treatment success By integrating Rhein’s intrinsic anticancer activity with the targeted delivery and controlled release afforded by selenium nanoparticle carriers, Rh-Se-NPs appear to exert prolonged cytotoxicity against cancer cells while mitigating systemic toxicity (Kuppusamy et al., 2016). This study did not include a direct comparison with standard colon-cancer therapeutics such as 5-fluorouracil. As such, any claims regarding therapeutic advantage or selectivity should be interpreted with caution. Future work incorporating clinical reference drugs will be essential for establishing comparative efficacy.

These visual morphological change findings align with the IC_50_ and selectivity index results, confirming that Rh-Se-NPs enhance the most pronounced anticancer activity while sparing normal cell morphology, supporting their potential as a targeted therapeutic option. The marked structural alterations observed in DLD-1 and SW620 cells after Rh-Se-NP treatment align with well-established morphological indicators of apoptosis, including cellular shrinkage, rounding, and detachment from the culture surface (Kroemer et al., 2007). Nanoparticle-based delivery systems can enhance the intracellular accumulation of anticancer agents and maintain effective concentrations over time, thereby intensifying morphological damage compared with free drug treatments (Blanco et al., 2015). Selenium nanoparticles, in particular, have been reported to promote reactive oxygen species (ROS) production and mitochondrial injury, leading to cytoskeletal disintegration and loss of cell adhesion in malignant cells (Albanese et al., 2012). In contrast, the absence of notable morphological changes in normal fibroblasts following treatment suggests a favorable therapeutic index. This selective toxicity is likely due to the greater metabolic activity and oxidative stress vulnerability of cancer cells relative to normal cells (Pelicano et al., 2004), in combination with the targeted delivery and sustained release properties of the Rh-Se-NP formulation (Kuppusamy et al., 2016). Collectively, these results indicate that Rh-Se-NPs not only suppress cell proliferation but also cause distinct structural degradation in colon cancer cells while preserving the architecture of healthy cells, underscoring their potential as selective anticancer agents.

Cell migration is a fundamental process in cancer metastasis, driven by cytoskeletal reorganization, degradation of the extracellular matrix (ECM), and epithelial–mesenchymal transition (EMT) (Lamouille et al., 2014). The marked reduction in migration observed with Rh-Se-NPs compared to free Rhein suggests that nanoparticle conjugation amplifies anti-metastatic activity through multiple mechanisms. Selenium nanoparticles have previously been shown to influence EMT marker expression, inhibit matrix metalloproteinase (MMP) activity, and disrupt focal adhesion kinase (FAK) signaling, collectively impairing cancer cell motility (Dai and Cai, 2021). The sustained presence of scratch gaps in Rh-Se-NP–treated cultures supports earlier findings that nanoparticle-based delivery systems can extend intracellular drug retention, maintain prolonged therapeutic levels, and enhance suppression of migration-related pathways (Lee, 2024). Rhein itself has been reported to inhibit EMT and restrict migration by downregulating Wnt/β-catenin and PI3K/AKT signaling (Fu et al., 2024). The superior effect of Rh-Se-NPs is likely attributable to improved cellular uptake and the synergistic redox-modulating properties of selenium, which can interfere with cytoskeletal dynamics critical for cell motility (Wadhwani et al., 2016). Overall, these findings indicate that Rh-Se-NPs suppress not only cancer cell proliferation, as demonstrated in IC_50_ assays, but also migration—a defining feature of metastatic progression—highlighting their promise as a dual-action therapeutic approach for colon cancer. Because proliferation was not inhibited or normalized during the wound-healing assay, part of the impaired wound closure may be attributable to reduced proliferation rather than decreased migration alone. Future studies using mitomycin-C or real-time migration tracking would help isolate the specific contribution of migration. IC_50_ concentrations was intended to provide a standardized comparison across assays, but that this approach does not isolate migration-specific effects. Future studies will employ sub-cytotoxic concentrations to definitively assess migration independent of cell viability. It should be noted that while normalization of wound width reduces variability, manual scratch assays remain subject to inherent limitations, which future studies may address through automated wound creation or advanced image analysis tools.

Caspase-3 functions as a principal executioner protease in apoptosis, orchestrating the final events such as DNA fragmentation and cytoskeletal breakdown (Elmore, 2007). In contrast, caspase-9 operates upstream as an initiator within the intrinsic apoptotic pathway, becoming activated following the release of cytochrome c from mitochondria under cellular stress (Li et al., 1997). The simultaneous elevation of both caspases observed here provides strong evidence for the activation of a mitochondria-dependent apoptotic cascade. Nanoparticle-based delivery platforms, including Rh-Se-NPs, can potentiate apoptosis by enhancing the solubility and bioavailability of hydrophobic drugs, promoting cellular internalization, and sustaining intracellular drug levels (Lee, 2024). Moreover, selenium nanoparticles have been shown to synergize with anticancer compounds by inducing oxidative stress and disrupting mitochondrial function, thereby increasing cancer cell susceptibility to apoptosis (Huang et al., 2019). Taken together, these findings indicate that Rh-Se-NPs function not only as carriers for Rhein but also as active co-therapeutic agents, enabling more efficient and robust activation of apoptotic pathways in colon cancer cells. It is important to note that although caspase-3 and caspase-9 activation strongly suggests engagement of apoptotic mechanisms, definitive confirmation of apoptosis can be further validated by other methods. Thus, our findings should be viewed as indicative of apoptotic involvement rather than conclusive proof.

PTEN, a tumor suppressor, was significantly upregulated, rising to 2.28 ± 0.15-fold with Rhein and an exceptional 15.9 ± 1.65-fold with Rh-Se-NPs, indicating a strong reactivation of anti-tumor signaling by the nanoparticle system. KRT18 and LGR6, associated with epithelial phenotype and cancer stemness, were substantially reduced by both treatments, with slightly higher residual expression in the Rh-Se-NP group, potentially reflecting differences in uptake or release kinetics. Interestingly, BCL2, an anti-apoptotic gene with low baseline expression (0.077 ± 0.002), was further reduced by Rhein (0.01 ± 0.001) but increased with Rh-Se-NPs (0.224 ± 0.003). This upregulation may represent a compensatory response to enhanced apoptotic stress induced by the nano-formulation and warrants further investigation. Overall, Rh-Se-NPs produced stronger tumor suppressor activation, maintained effective oncogene repression, and offered nuanced regulation of angiogenic and apoptotic pathways, reinforcing their promise as an improved therapeutic approach for metastatic colon cancer. Overall, both formulations suppressed oncogenic and angiogenic gene expression in the two investigated cancer cells (DLD-1 and SW620), while enhancing tumor-suppressive signaling, with Rh-Se-NPs generally achieving stronger or more balanced modulation, underscoring their potential as an improved therapeutic option for colon cancer. The observed gene expression changes indicate that Rh-Se-NPs mediate their anticancer effects through a concerted inhibition of oncogenic signaling and activation of tumor-suppressive pathways. The marked elevation of PTEN, a pivotal modulator of the PI3K/AKT pathway, suggests restoration of apoptotic signaling, leading to reduced cell proliferation and enhanced programmed cell death (Song et al., 2012). The moderate suppression of VEGFA by Rh-Se-NPs may provide dual benefits, attenuating angiogenesis while avoiding adverse outcomes associated with excessive vascular inhibition (Ferrara and Adamis, 2016; Sitohy et al., 2017). The pronounced downregulation of LGR6, a marker linked to cancer stemness, implies interference with stem cell–like properties, which are known to contribute to therapy resistance and metastatic potential (de Lau et al., 2007). The increase in BCL2 expression observed in SW620 cells could represent a transient adaptive response to apoptotic stress triggered by greater nanoparticle internalization; however, the overall pro-apoptotic context, evidenced by elevated caspase activity, indicates that cell death pathways remain dominant (Czabotar et al., 2014). Collectively, these findings suggest that Rh-Se-NPs not only enhance Rhein delivery but also optimize its molecular effects, thereby achieving robust anticancer activity with the potential for an improved safety profile through simultaneous modulation of multiple pathways.

The gene-expression patterns observed in Table 9 include some seemingly contradictory findings, such as the upregulation of BCL2 in SW620 cells and the more pronounced VEGFA suppression induced by free rhein compared to its conjugates. These variations likely reflect the inherent biological differences between the tested cell lines and the context-dependent regulation of apoptotic and angiogenic pathways. In SW620, BCL2 upregulation may represent a compensatory survival response triggered by treatment-induced stress, rather than a direct anti-apoptotic effect. Conversely, the stronger VEGFA suppression with free rhein may be attributed to its higher cellular uptake or faster intracellular availability compared with conjugated forms. Therefore, these findings should not be viewed as contradictions but rather as indicators of the complexity and cell-specificity of the signaling responses to rhein-based treatments. Taken together, the gene-expression outcomes reveal a multifaceted regulatory response rather than a uniform linear effect, underscoring that rhein and its conjugates act through interconnected pathways whose directionality may vary across cell-type-specific regulatory programs. Overall, while the treatments produced clear caspase activation and measurable reductions in wound closure, these outcomes should be interpreted cautiously, given the study’s methodological limitations. An important limitation of the present study is the absence of direct apoptosis assays, although caspase activation and gene modulation strongly support apoptotic involvement. Hence, definitive confirmation will require employing direct methods in future studies. The present results provide mechanistic indications rather than definitive mechanistic confirmation.

## 5 Conclusion

The present study successfully demonstrated the green synthesis, characterization, and enhanced anticancer potential of Rhein-mediated selenium nanoparticles (Rhein-NPs). Rhein was effectively isolated and confirmed via ^1^H and ^13^C NMR spectroscopy, exhibiting high purity. The Rhein-NPs showed uniform spherical morphology with an average diameter of ∼32 nm (TEM), hydrodynamic size of ∼90 nm (DLS), and high stability with a zeta potential of −31.1 mV. Crystallinity was confirmed by XRD, revealing face-centered cubic selenium with an average crystallite size of 28.3 nm. Biological assays revealed that Rhein-NPs significantly enhanced cytotoxicity against DLD-1 and SW620 colon cancer cells compared to free Rhein, while sparing normal FSU fibroblast cells. The nanoformulation improved IC_50_ values and selectivity indices, indicating enhanced efficacy and safety. Rhein-NPs induced greater morphological disruption, migration inhibition, and caspase-3/9 activation, supporting strong apoptotic activity. Gene expression analysis showed superior modulation by Rhein-NPs, notably upregulating tumor suppressor PTEN and downregulating oncogenic markers (CEA, FOXQ1, VEGFA). Overall, Rhein-NPs demonstrated superior physicochemical properties and potent anticancer activity through apoptosis supported by caspase activation and gene regulation. These findings underscore the potential of Rhein-NPs as a promising, biocompatible therapeutic strategy for targeted colon cancer treatment. Further *in vivo* and mechanistic studies are warranted.

The novelty of this work lies in the phytochemical-guided green synthesis of selenium nanoparticles with dual apoptotic and gene-modulatory actions. The significant upregulation of PTEN, alongside the suppression of oncogenes such as CEA, FOXQ1, and VEGFA, demonstrates the ability of Rhein-NPs to target key molecular pathways in colon cancer. Given their selective cytotoxicity and stability, Rhein-NPs offer a compelling direction for the development of next-generation, eco-friendly nanotherapeutics with translational potential in oncology.

While the present study demonstrates that Rh-Se-NPs significantly impair colon cancer cell viability, induce caspase activation, and modulate oncogenic gene expression, the antimigration findings should be interpreted with caution. Because wound-healing assays were conducted at IC_50_ concentrations, the observed reduction in wound closure may reflect combined effects of cytotoxicity and impaired migration rather than migration-specific inhibition. Future investigations using sub-cytotoxic concentrations and/or proliferation inhibitors, together with concurrent viability measurements, will be essential to definitively isolate migration effects. This limitation does not diminish the overall significance of the dual-action anticancer potential demonstrated here, but it does highlight the need for further refinement in experimental design to strengthen mechanistic conclusions.

## Data Availability

The raw data supporting the conclusions of this article will be made available by the authors, without undue reservation.
