# Correction: Green-synthesized rhein-selenium nanoparticles exhibit potent and highly selective anticancer activity against colon cancer via apoptosis and gene regulation

**DOI:** 10.3389/fchem.2026.1960735

**Published:** 2026-08-27

**Authors:** Mohamed D. Abd El-Halim, Ali Osman, Marwa A. Ibrahim, Mayada M. El-Azab, Mahmoud M. El-Saber, Sameh H. Ismail, Tamer Roshdy, Mahmoud Sitohy, Basel Sitohy

**Affiliations:** 1 Medicinal and Aromatic Plants Department, Desert Research Center, Cairo, Egypt; 2 Department of Clinical Microbiology, Infection, and Immunology, Umeå University, Umeå, Sweden; 3 Department of Diagnostics and Intervention, Oncology, Umeå University, Umeå, Sweden; 4 Department of Biochemistry, Faculty of Agriculture, Zagazig University, Zagazig, Egypt; 5 Genetic Resources Department, Biochemistry Unit, Desert Research Center, Cairo, Egypt; 6 Faculty of Nanotechnology for Postgraduate Studies, Cairo University, Sheikh Zayed Campus, Giza, Egypt; 7 Department of Molecular Biology, Faculty of Biotechnology, University of Sadat City, Sadat, Egypt

**Keywords:** apoptosis induction, caspase-3 and caspase-9 activation, colon cancer therapy, gene expression modulation, green synthesis, oncogene suppression, rhein-selenium nanoparticles

There was a mistake in the caption of **Figure 3** as published. There were missing values. The corrected caption appears below.

FIGURE 3 | Comprehensive characterization of Rhein-mediated selenium nanoparticles (Rhein-SeNPs). **(A)** Transmission electron microscopy (TEM) image showing spherical nanoparticles with uniform distribution and an average diameter of 32 ± 5 nm. **(B)** Atomic force microscopy (AFM) confirming the nanoscale height profile and surface morphology. **(C)** Dynamic light scattering (DLS) analysis indicating an average hydrodynamic size of 90.4 nm with a narrow size distribution. **(D)** Zeta potential measurement showing a surface charge of −31.1 mV, suggesting good colloidal stability. **(E)** X-ray diffraction (XRD) pattern confirming the crystalline/semi-crystalline nature of the synthesized SeNPs.

The two mentions of XPS were inadvertent errors.

A correction has been made to section **1 Introduction**, paragraph 7. The corrected sentence reads:

“Our formulation advances the field through four distinct innovations: first, employing pure, NMR-characterized rhein rather than crude extracts; and second, ensuring reproducible synthesis and defined molecular interactions, as confirmed by FTIR spectroscopy.”

A correction has been made to section **3 Results**, “3.1 Characterization of rhein and Rh-Se-NPs,” paragraph 16. The corrected sentence reads:

“These STEM-EDX findings provide spatial confirmation of core-shell architecture with selenium core and rhein organic shell, validating the proposed nanoparticle structure and complementing the FTIR surface characterization.”

The value 10^4^ was incorrectly displayed as 10^4, and the words “from soybean, chickpea, and lupin” were erroneously included. A correction has been made to section **2 Materials and methods**, “2.7 *In vitro* cytotoxicity”, *2.7.2 MTT-assay*, paragraph 1. The corrected sentence reads:

“Cells 1.0 × 10^4^ were seeded in 96-well plates for 24 h, then treated with Rhein or Rhein-NPs (150 μL/well) at concentrations of 800 to 25 μg/mL; controls received PBS.”

